# Methionine restriction-induced sulfur deficiency impairs antitumour immunity partially through gut microbiota

**DOI:** 10.1038/s42255-023-00854-3

**Published:** 2023-08-03

**Authors:** Ming Ji, Xiaojiang Xu, Qing Xu, Yun-Chung Hsiao, Cody Martin, Svetlana Ukraintseva, Vladimir Popov, Konstantin G. Arbeev, Tom A. Randall, Xiaoyue Wu, Liz M. Garcia-Peterson, Juan Liu, Xin Xu, M. Andrea Azcarate-Peril, Yisong Wan, Anatoliy I. Yashin, Karthik Anantharaman, Kun Lu, Jian-Liang Li, Igor Shats, Xiaoling Li

**Affiliations:** 1 Signal Transduction Laboratory, National Institute of Environmental Health Sciences, Research Triangle Park, NC USA; 2 Integrative Bioinformatics, National Institute of Environmental Health Sciences, Research Triangle Park, NC USA; 3 Department of Environmental Sciences and Engineering, Gillings School of Global Public Health, University of North Carolina at Chapel Hill, Chapel Hill, NC USA; 4 Department of Bacteriology, University of Wisconsin-Madison, Madison, WI USA; 5 Microbiology Doctoral Training Program, University of Wisconsin–Madison, Madison, WI USA; 6 Social Science Research Institute, Duke University School of Medicine, Durham, NC USA; 7 Department of Pharmacology and Cancer Biology, Duke University School of Medicine, Durham, NC USA; 8 Epigenetics and Stem Cell Biology Laboratory, National Institute of Environmental Health Sciences, Research Triangle Park, NC USA; 9 Department of Medicine, Division of Gastroenterology and Hepatology and Microbiome Core Facility, University of North Carolina at Chapel Hill, Chapel Hill, NC USA; 10 Department of Microbiology and Immunology and Lineberger Comprehensive Cancer Center, University of North Carolina at Chapel Hill, Chapel Hill, NC USA

**Keywords:** Cancer microenvironment, Cancer immunotherapy, Metabolism, Microbiome

## Abstract

Restriction of methionine (MR), a sulfur-containing essential amino acid, has been reported to repress cancer growth and improve therapeutic responses in several preclinical settings. However, how MR impacts cancer progression in the context of the intact immune system is unknown. Here we report that while inhibiting cancer growth in immunocompromised mice, MR reduces T cell abundance, exacerbates tumour growth and impairs tumour response to immunotherapy in immunocompetent male and female mice. Mechanistically, MR reduces microbial production of hydrogen sulfide, which is critical for immune cell survival/activation. Dietary supplementation of a hydrogen sulfide donor or a precursor, or methionine, stimulates antitumour immunity and suppresses tumour progression. Our findings reveal an unexpected negative interaction between MR, sulfur deficiency and antitumour immunity and further uncover a vital role of gut microbiota in mediating this interaction. Our study suggests that any possible anticancer benefits of MR require careful consideration of both the microbiota and the immune system.

## Abstract (web summary)

Dietary methionine restriction has been reported to protect from cancer. Ji et al. describe a cancer-promoting effect of methionine restriction mediated by gut microbiota-induced sulfur deficiency and suppression of antitumour immunity.

## Main

Methionine, a sulfur-containing essential amino acid, is a key component of dietary proteins important for protein synthesis, sulfur metabolism, epigenetic modification, antioxidant defence and signalling^1^. However, the role of methionine in regulating cancer progression remains inconclusive. On one hand, many cancer cells, including tumour-initiating cells and HNF4α-positive liver cancer cells, highly depend on exogeneous methionine^2,3^. MR, a dietary regimen that protects against metabolic diseases and ageing^4,5^, is known to repress the proliferation and progression of various xenograft tumours^3,6–9^. This dietary intervention can impact metabolic flux in one-carbon metabolism, inhibit tumour growth and sensitize tumours to chemotherapy and radiotherapy in certain patient-derived xenografts (PDXs) in a tumour cell-autonomous manner^10^. On the other hand, methionine is also critical for T cell activation and differentiation^11,12^. Competition for methionine between fast-growing tumour cells and T cells in the tumour microenvironment has been reported to disrupt methionine metabolism inside CD8^+^ T cells, lowering intracellular levels of methionine and the methyl donor *S*-adenosylmethionine (SAM)^13^. This reduction leads to loss of dimethylated histone H3 Lys 79 (H3K79me2) mark, decreased expression of STAT5, and impairment of T cell immunity and checkpoint-induced tumour immunity^13^. Therefore, methionine is also a potential tumour-suppressing nutrient that enhances T cell-mediated antitumour immunity. Here, we investigated the interaction between dietary methionine, immune cells and cancer cells in immunocompetent mice.

## Low dietary methionine or protein intake is associated with aggravated tumour progression in mice and increased cancer risk in humans

**Fig. 1:**
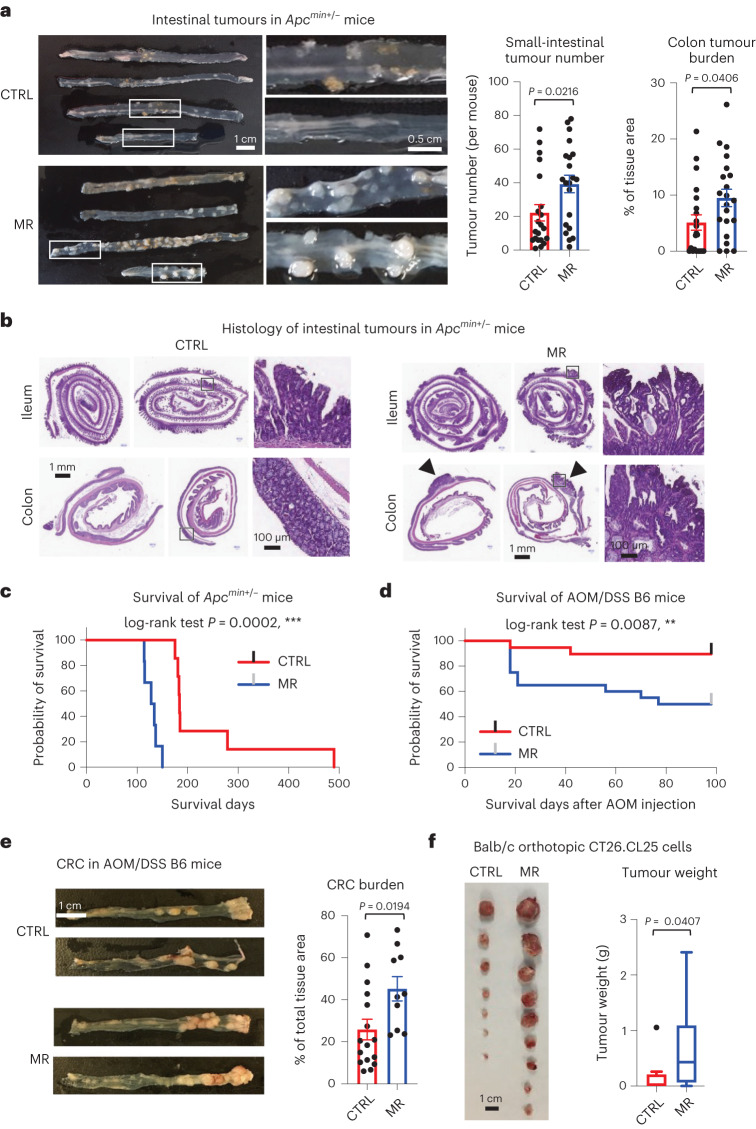
Methionine restriction enhances tumour progression in immunocompetent mice. **a**, MR increased intestinal tumour growth in *Apc*^*min+/−*^ mice (*n* = 21 mice per group, two-tailed unpaired Student’s *t*-test; values are expressed as mean ± s.e.m.). **b**, Representative images of H&E-stained ileum and colon sections. Arrowheads, colonic tumours. **c**, MR reduced the survival of *Apc*^*min+/−*^ mice (*n* = 7 mice on CTRL diet and 6 mice on MR diet, log-rank test). **d**, MR sensitized C57BL/6 mice to AOM/DSS-induced death. Regular B6 mice were subjected to a modified AOM/DSS CRC procedure as described in Methods (*n* = 19 CTRL and 20 MR initial mice, log-rank test). **e**, MR pre-feeding enhanced tumour progression in C57BL/6 mice in an AOM/DSS CRC model (*n* = 16 mice on CTRL diet and 10 mice on the MR diet, two-tailed unpaired Student’s *t*-test; values are expressed as mean ± s.e.m.). **f**, MR pre-feeding enhanced the growth of orthotopically implanted CT26.CL25 cells in Balb/c mice (*n* = 10 tumours per group, two-tailed unpaired Student’s *t*-test, one outlier in the control group was removed by >Q3 + 3.0 times the interquartile range (IQR); box-and-whiskers plot, Tukey with whiskers: Q1 minus 1.5 times the IQR to Q3 plus 1.5 times the IQR). Details of statistical tests are in Methods. Source data

**Extended Data Fig. 1:**
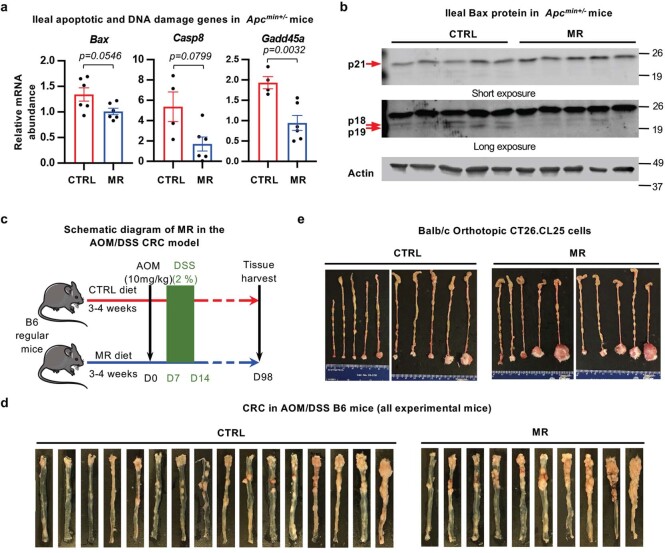
Dietary methionine restriction enhances tumor progression in immunocompetent mice. **a**, MR-induced tumor growth is associated with reduced expression of apoptotic and DNA damage response genes in the ileum of *Apc*^*min+/−*^mice. The expression of indicated apoptotic genes was analyzed by qPCR (For *Bax*, n = 6 mice on CTRL diet; 6 mice on MR diet; For *Casp8* and *Gadd45a*, n = 4 mice on CTRL diet; 6 mice on MR diet; two-tailed unpaired Student’s t-test. Values are expressed as mean ± s.e.m.). **b**, MR increases the cleavage of Bax in the ileum of *Apc*^*min+/−*^ mice. The ileum sections from mice fed with CTRL diet and MR diet were immunoblotted with an anti-Bax antibody. The ilea from five pairs of CTRL and MR diet fed *Apc*^*min+/−*^ mice from one experiment were analyzed. **c**, Schematic diagram of dietary methionine restriction in the AOM/DSS CRC model. Regular C57BL/6 mice (B6) were fed with CTRL diet or MR diet for 3-4 weeks. They were then i.p. Injected with 10 mg/kg bwt AOM. One week later, they were treated with 2 % DSS in drinking water for 7 days, then return to regular water for the rest of the procedure. Colon tissues were dissected 14 weeks after the AOM injection to analyze the development and progression of CRC. Mouse cartoon images were drawn by using pictures from Servier Medical Art (https://smart.servier.com/wp-content/uploads/2016/10/Animals.pptx). Servier Medical Art by Servier is licensed under a Creative Commons Attribution 3.0 Unported License (https://creativecommons.org/licenses/by/3.0/). **d**, MR enhances tumor progression in C57BL/6 mice in an AOM/DSS CRC model. B6 mice were treated as in (**c**). Colon images from all experimental mice are shown. **e**, MR enhances orthotopically grafted CT26.CL25 tumor growth. Images of orthotopic tumors are shown. Details of statistical tests are included in the Methods. Source data

To investigate the potential impact of dietary methionine on tumour progression, we fed a genetic intestinal tumour model, *Apc*^*min+/−*^ mice^14,15^, with either a methionine-restricted diet (MR diet) containing 0.172% dl-methionine or a control diet (CTRL diet) containing 0.86% dl-methionine starting from the age of 2 months. Both diets were cystine free. Since a number of comparable methionine-restricted diets have been shown to suppress a variety of xenograft tumours^6–9^, including colorectal PDX tumours in mice^10^, we were surprised to find that *Apc*^*min+/−*^ mice fed with the MR diet had a significantly higher number of tumours in the small intestine and a higher tumour burden in the colon (Fig. 1a,b), along with a dramatically shortened symptom-free survival (Fig. 1c), compared to those fed with the control diet. Additionally, the ileum of the MR diet-fed mice had reduced expression levels of several apoptotic and DNA damage response genes (Extended Data Fig. 1a) and decreased cleavage of apoptotic protein Bax (Extended Data Fig. 1b), indicating that intestinal tumours developed on the MR diet are more resistant to cell death than those developed on the control diet.

To exclude possible cancer model-specific impacts of the MR diet on intestinal tumour growth, we performed dietary MR in C57BL/6J (B6) mice under an azoxymethane (AOM)/dextran sodium sulfate (DSS)-induced colorectal cancer (CRC) procedure (Extended Data Fig. 1c). Consistent with our observations in *Apc*^*min+/−*^ mice, pre-feeding with the MR diet for 3–4 weeks substantially reduced the survival of B6 animals during the AOM/DSS procedure (Fig. 1d) and significantly increased the CRC burden in the surviving mice at the end stage (Fig. 1e and Extended Data Fig. 1d). Moreover, in a syngeneic colon cancer model, MR pre-feeding significantly enhanced the growth of orthotopically (rectum) implanted CT26.CL25 mouse colon carcinoma cells in Balb/c mice (Fig. 1f and Extended Data Fig. 1e).

Importantly, in a collaborative supplementary study, we found the inverse relationship between the dietary protein intake (proxy for methionine intake) and cancer risk in humans, using a subset of the UK Biobank (UKB) participants (31,626 total, 52% females). The daily protein intake was categorized as relatively low (lowP; <1.0 g per kg body weight per day) versus high (highP; >1.6 g per kg body weight per day). Combined (all) and male only participants in the lowP group had significantly higher overall cancer risk compared to those in the highP group (Supplementary Table 1). The low dietary methionine/protein intake was, therefore, unexpectedly associated with cancer development/progression in both mice and humans in our study.

## Methionine restriction decreases T cell abundance and reduces the efficacy of antitumour immunotherapy in immunocompetent mice

**Fig. 2:**
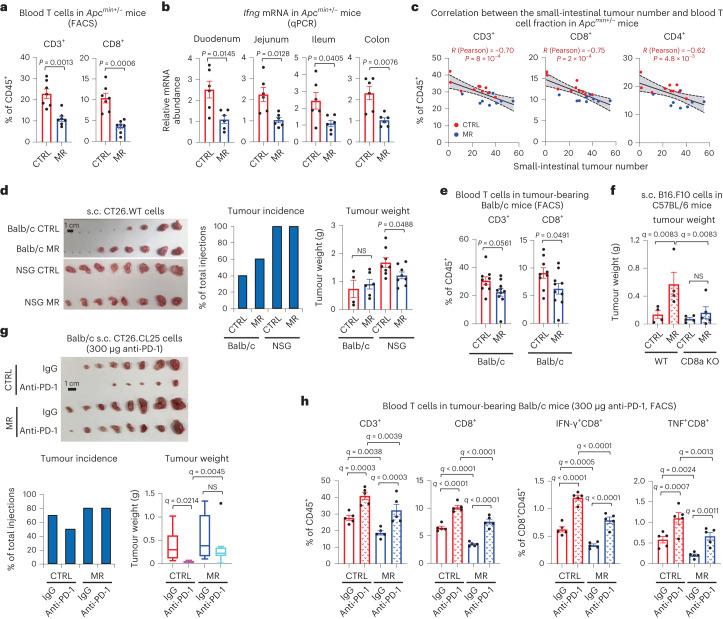
Methionine restriction represses T cell activation and blunts tumour response to anticancer immunotherapy in immunocompetent mice. **a**, MR reduced the fraction of circulating T cells in *Apc*^*min+/−*^ mice (*n* = 7 mice per group, two-tailed unpaired Student’s *t*-test). **b**, MR reduced the expression of *Ifng* in all segments of the tumour-containing intestine of in *Apc*^*min+/−*^ mice (*n* = 6 mice per group, two-tailed unpaired Student’s *t*-test). **c**, The small-intestinal tumour number was negatively correlated with the abundance of circulating T cells in *Apc*^*min+/−*^ mice fed with CTRL or MR diets. One-month-old *Apc*^*min+/−*^mice were fed with CTRL or MR diet for 3 months (*n* = 10 mice for CTRL diet, 9 mice for MR diet). The Pearson correlation coefficient was analysed in Prism (two tailed, 95% confidence intervals are labelled). **d**, The impact of MR on tumour incidence and growth was dependent on the status of host immune system (*n* = 10 tumours per group for Balb/c mice; 8 tumours/for NSG mice, two-tailed unpaired Student’s *t*-test within each mouse strain). **e**, MR reduced blood CD3^+^ T cells in tumour-bearing immunocompetent Balb/c mice. The percentage of the indicated circulating T cell populations from Balb/c mice in **d** was analysed by flow cytometry (*n* = 10 mice per group, two-tailed unpaired Student’s *t*-test). **f**, MR failed to enhance the growth of allografted B16.F10 mouse melanoma cells in CD8a knockout mice (for WT mice, *n* = 4 mice per group; for CD8a knockout mice, *n* = 5 mice per group; two-way ANOVA). **g**, Anti-PD-1 antibody (300 μg per injection) failed to suppress tumour growth in methionine-restricted immunocompetent mice (*n* = 7 tumours for CTRL IgG group; 5 tumours for CTRL anti-PD-1 group; 8 tumours for MR IgG group; and 8 tumours for MR anti-PD-1 group; two-way ANOVA, one outlier in the MR anti-PD-1 group was removed by >Q3 + 3.0 times the IQR; box-and-whiskers plot, Tukey with whiskers: Q1 minus 1.5 times the IQR to Q3 plus 1.5 times the IQR). Mice were inoculated with tumour cells in both flanks. **h**, MR reduced circulating CD3^+^ and CD8^+^ T cells in tumour-bearing Balb/c mice treated with or without 300 μg per injection of anti-PD-1 (*n* = 5 mice per group, two-way ANOVA). Values are expressed as the mean ± s.e.m., except in **c**. Details of statistical tests are in Methods. NS, not significant. Source data

**Extended Data Fig. 2:**
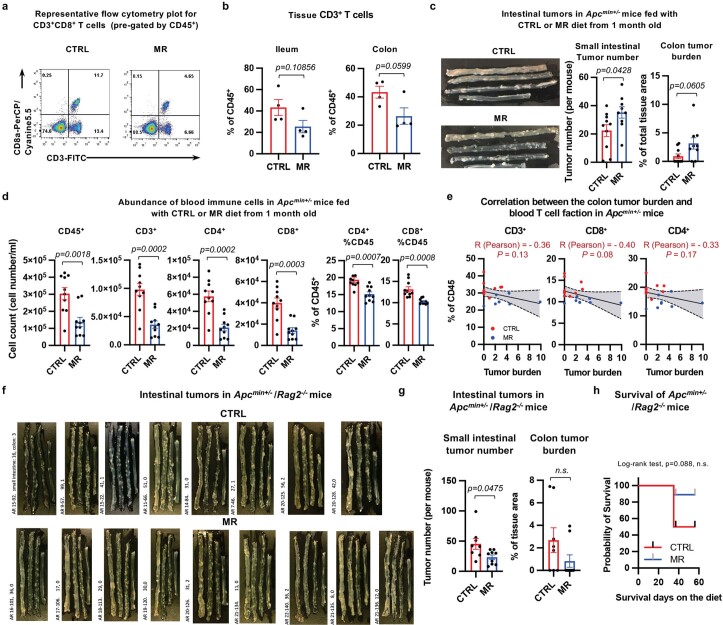
Dietary methionine restriction enhances tumor progression in immunocompetent *Apc*^*min+/−*^ mice by modulating anti-tumor immunity. **a**, MR reduces the fraction of CD3^+^CD8^+^ T cells in the blood of *Apc*^*min+/−*^ mice. Representative flow cytometry plots are shown. **b**, MR has a trend to reduce the fraction of CD3^+^ T cells in intestinal tissues of *Apc*^*min+/−*^ mice (n = 4 mice in each group, two-tailed unpaired Student’s t-test). **c**, MR from age of one month increases tumor growth in the intestine of *Apc*^*min+/−*^mice. One-month old *Apc*^*min+/−*^ mice were fed with with CTRL or MR diet for 3 months (n = 10 mice for CTRL diet, 9 mice for MR diet, two-tailed unpaired Student’s t-test). **d**, MR from age of one month reduces blood T cells abundance in *Apc*^*min+/−*^mice. *Apc*^*min+/−*^mice were fed as in (**c**) and the cell number of indicated cells in the blood was analyzed by FACS (n = 10 mice in each diet, two-tailed unpaired Student’s t-test). **e**, Correlation between colon tumor burden and blood T cell faction of *Apc*^*min+/−*^ mice fed with CTRL or MR diet for 3 months (n = 10 mice for CTRL diet, 9 mice for MR diet). The Pearson correlation coefficient between the tumor burden in the colon with the blood fraction of indicated T cells was analyzed in Prism. **f–g**, MR suppresses intestinal tumor growth in *Apc*^*min+/−*^ mice on an immunodeficient background. *Apc*^*min+/−*^ /*Rag2*^*−/−*^ mice were fed with CTRL or MR diet for about 2 months. Their small intestinal tumor number and colon tumor burden were analyzed around 4 months of age (n = 8 mice on the CTRL diet and 9 on the MR diet, two-tailed unpaired Student’s t-test). **h**, Healthy survival of *Apc*^*min+/−*^ /*Rag2*^*−/−*^ mice under CTRL or MR diet (n = 8 mice on the CTRL diet and 9 on the MR diet, log-rank test). Values are expressed as mean ± s.e.m., except (**e** and **h**). Details of statistical tests are included in the Methods. Source data

Mice used in the above three different intestinal/colon cancer models, including *Apc*^*min+/−*^ mice, C57BL/6J mice and Balb/c mice, are all immunocompetent in contrast with immunodeficient mice utilized in previous xenograft and PDX studies^6–10^. We thus tested whether MR impairs T cell differentiation and activation, which are known to be supported by methionine^11–13^, in these immunocompetent mice. Indeed, in *Apc*^*min+/−*^ mice, MR diet feeding significantly reduced the fraction of circulating CD3^+^ and CD8^+^ T cells (Fig. 2a and Extended Data Fig. 2a). It also displayed a trend to decrease the abundance of CD3^+^ T cells in the ileum and colon (Extended Data Fig. 2b) and significantly repressed the expression of *Ifng*, which encodes interferon (IFN)-γ, a key tumour immune surveillance cytokine predominantly produced by activated lymphocytes^16^, in all segments of the intestine of MR diet-fed *Apc*^*min+/−*^ mice (Fig. 2b). Dietary MR in *Apc*^*min+/−*^ mice from the age of 1 month, before any visible intestinal tumours were developed, also enhanced tumour progression (Extended Data Fig. 2c). Strikingly, in these MR diet-fed *Apc*^*min+/−*^ mice, the total cell counts of circulating CD45^+^ immune cells were dramatically reduced in addition to the cell counts and fractions of T cells (Extended Data Fig. 2d). Moreover, the fractions of their circulating T cells were negatively correlated with the intestinal tumours, particularly the number of small-intestinal tumours (Fig. 2c and Extended Data Fig. 2e). All these observations suggest that MR promotes intestinal tumour progression by hindering overall immune cell differentiation, proliferation and/or survival in this tumour model. In support of this possibility, MR failed to promote the intestinal tumour number/burden in *Apc*^*min+/−*^ mice on an immunodeficient background (*Rag2*^*−/−*^; Extended Data Fig. 2f–h).

**Extended Data Fig. 3:**
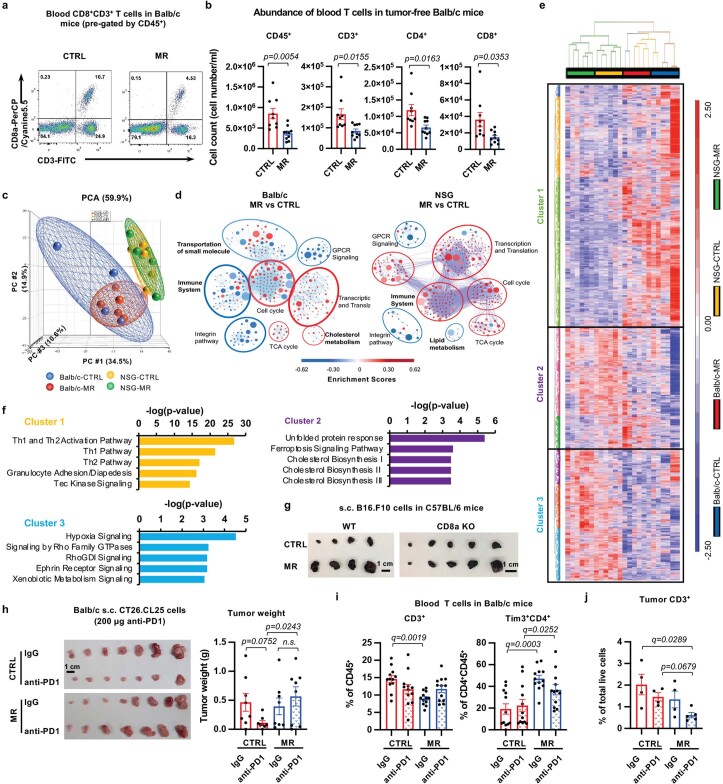
MR suppresses anti-tumor immunity and enhances tumor progression in syngeneic cancer models. **a**, Representative flow cytometry plots of blood CD8^+^CD3^+^ T cells in tumor-bearing Balb/c mice. **b**, MR reduces blood CD3^+^ T cells in tumor-free Balb/c mice (n = 9 mice/group, two-tailed unpaired Student’s t-test). **c**, MR exerts distinct impacts on the transcriptomic profiles of CT26.WT tumors in Balb/c mice or NSG mice. The transcriptomes of CT26.WT tumors collected from indicated mice were analyzed by RNA-seq (n = 6 tumors in each group). **d**, GSEA pathway enrichment maps for MR-induced gene sets in CT26/WT tumors collected in Balb/c mice or NSG mice. Enrichment maps were generated using Cytoscape. For each node, size represents “size of gene set”, the density of color represents “Enrichment score. **e**, The mRNA levels of 8,233 significantly differentially expressed genes from (**c**) were clustered and presented by heatmaps (Cluster 1, 4031; Cluster 2, 2041; Cluster 3, 2151). **f**, The top five enriched canonical pathways in the three gene clusters in (**e**) identified by Ingenuity Pathway Analysis. **g**, MR fails to enhance the growth of B16.F10 mouse melanoma cells in CD8a KO mice. WT C57BL/6 control mice and CD8a KO mice were fed with CTRL or MR diets. B16.F10 mouse melanoma cells were injected and the image for final tumors are shown. **h**, Anti-PD-1 antibody (200 µg/injection) fails to suppress tumor growth in methionine-restricted immunocompetent mice (n = 8 tumors/group, 2-way ANOVA). **i**, MR reduces circulating CD3^+^ T cells but increases circulating Tim3^+^CD4^+^ exhausted T cells in Balb/c mice (n = 12 mice/group, 2-way ANOVA). **j**, MR reduces tumor CD3^+^ T cells in anti-PD-1 antibody treated mice (n = 4 tumors for CTRL IgG group; 4 tumors for CTRL anti-PD-1 group; 4 tumors for MR IgG group; and 5 tumors for MR anti-PD-1 group, 2-way ANOVA). Values are expressed as mean ± s.e.m. Details of statistical tests are included in the Methods. Source data

To directly evaluate the role of the immunity in modulating the effects of MR on tumours, we compared the effects of MR diet feeding on the incidence and growth of subcutaneously (s.c.) grafted CT26 wild-type (WT) mouse colon carcinoma cells in immunocompetent Balb/c mice and immunocompromised NSG mice^17^. Consistent with previous xenograft reports^3,6–9^, MR diet feeding suppressed the growth of subcutaneously inoculated CT26.WT tumours in immunocompromised NSG mice (Fig. 2d). However, this suppression was lost in immunocompetent Balb/c mice (Fig. 2d). Importantly, even though MR diet pre-feeding did not significantly enhance the growth of subcutaneously inoculated CT26 tumours as it did for orthotopically implanted CT26 tumours (Fig. 1f), it increased the tumour incidence (Fig. 2d). Moreover, consistent with the observations from *Apc*^*min+/−*^ mice (Fig. 2a and Extended Data Fig. 2a), MR also decreased the circulating fractions of CD3^+^ and CD8^+^ T cells in above tumour-bearing Balb/c mice (Fig. 2e and Extended Data Fig. 3a), and substantially reduced the abundance of circulating CD45^+^ immune cells, including T cells in tumour-free regular Balb/c mice (Extended Data Fig. 3b). Further RNA-sequencing (RNA-seq) analysis confirmed that subcutaneously grafted CT26.WT tumours responded distinctly at the transcriptomic level to MR in Balb/c and NSG mice (Extended Data Fig. 3c–f and Supplementary Tables 2 and 3), indicating that host immune activity is a key determinant of the tumour response to MR.

All the above observations strongly suggest that MR enhances tumour growth and progression in immunocompetent mice by reducing immune cells, particularly T cells. In agreement with this possibility, pre-feeding of the MR diet increased the growth of B16.F10 melanoma tumours subcutaneously grafted in WT C57BL/6J mice but not in CD8a knockout C57BL/6J mice (Fig. 2f and Extended Data Fig. 3g). Furthermore, when used at a dose of 300 μg per injection for each mouse, anti-PD-1 (an immune checkpoint inhibitor (ICI) targeting T cells), significantly suppressed the growth and incidence of subcutaneously grafted CT26.CL25 cells, a beta-galactosidase expressing subclone of CT26.WT widely used for testing immunotherapy protocols and host immune response^18^, in control diet-fed but not MR diet-fed Balb/c mice (Fig. 2g and Supplementary Fig. 1a), indicating that MR reduces the efficacy of ICIs. Consistently, although the abundance and activation of circulating T cells were significantly induced by this anti-PD-1 dose in mice fed with both diets, they were significantly lower in mice on the MR diet compared to those in mice on the CTRL diet (Fig. 2h). A lower dose (200 μg per injection for each mouse) of anti-PD-1 treatment also suppressed the growth of CT26.CL25 tumours in Balb/c mice on the control diet but not on MR diet (Extended Data Fig. 3h). In these MR-fed tumour-bearing Balb/c mice, the proportion of CD3^+^ T cells was reduced, while that of Tim3^+^CD4^+^ exhausted T cells was increased in the blood (Extended Data Fig. 3i and Supplementary Fig. 1b,c). Their intratumoural CD3^+^ T cells also showed a trend of reduction after the anti-PD-1 treatment (Extended Data Fig. 3j and Supplementary Fig. 1d). Taken together, our findings demonstrate that MR represses T cells and dampens the efficacy of ICI-mediated antitumour therapy in immunocompetent mice.

## Methionine restriction promotes intestinal cancer progression and suppresses antitumour immunity through gut microbiota

Methionine has been reported to promote T cell activation and differentiation through a SAM-mediated cell-autonomous enhancement of histone activation marks, such as trimethylated histone H3 Lys4 (H3K4me3) on the promoters of genes involved in cytokine production and cell cycle progression^11,12^ or H3K79me2 on key immunoregulatory transcription factor STAT5 (ref. ^13^). However, given that the gut microbiota plays a fundamental role in regulating the host immune system^19^ and the efficacy of antitumour immunotherapies^20^, we hypothesized that in addition to its direct effects on the immune cells, dietary methionine might affect systemic immune function, antitumour immunity and outcomes of antitumour immunotherapy through modulating the gut microbiota.

**Fig. 3:**
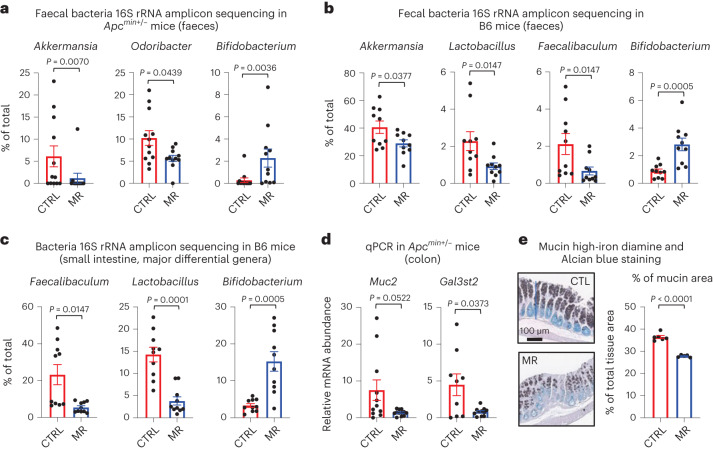
Methionine restriction alters gut microbiota in immunocompetent mice. **a**, MR significantly disrupted three major bacterial taxa in *Apc*^*min+/−*^ mice. The faecal bacteria abundance in diet-fed *Apc*^*min+/−*^ mice were determined by 16S rRNA gene amplicon sequencing (*n* = 12 mice on CTRL diet; 11 mice on MR diet, two-tailed unpaired Student’s *t*-test). **b**, MR significantly disrupted four major bacterial groups in C57BL/6J (B6) mice (*n* = 10 mice per group, two-tailed unpaired Student’s *t*-test). **c**, MR reduced *Faecalibaculum* and *Lactobacillus* but increased *Bifidobacterium* in the small intestine of B6 mice (*n* = 10 mice per group, two-tailed unpaired Student’s *t*-test). **d**, MR repressed the expression of *Muc2* and *Gal3st2* in the tumour-containing colons of *Apc*^*min+/−*^ mice. The mRNA abundance of *Muc2* and *Gal3st2* was analysed by qPCR with lamin as a loading control (*n* = 6 mice on CTRL diet and 5 mice on MR diet, two-tailed unpaired Student’s *t*-test; one outlier in *Gal3st2* MR group was removed by >Q3 + 3.0 times the IQR). **e**, MR reduced the mucin in the colons of *Apc*^*min+/−*^ mice. The colonic sections of *Apc*^*min+/−*^ mice on different diets were stained with the high-iron diamine and Alcian blue, and the percentages of total mucin-positive area were quantified in Fiji (*n* = 6 mice on control diet and *n* = 5 mice on MR diet, two-tailed unpaired Student’s *t*-test). Values are expressed as the mean ± s.e.m. Details of statistical tests are in Methods. Source data

**Extended Data Fig. 4:**
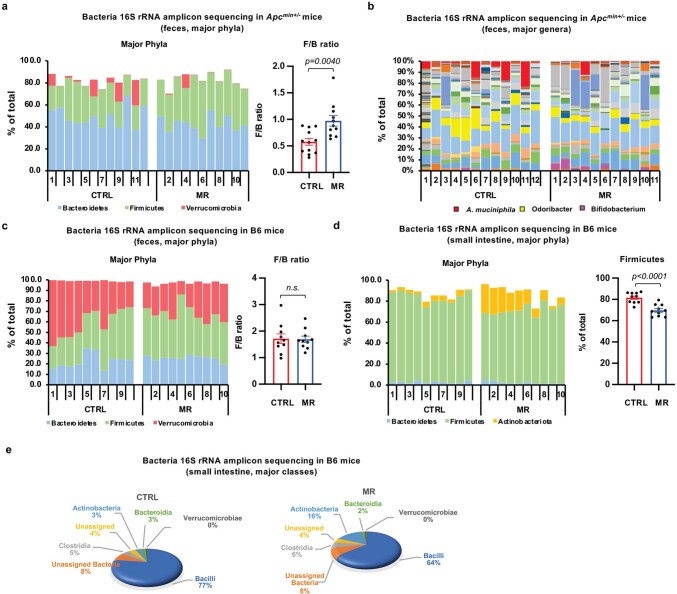
Dietary methionine restriction alters gut microbiota in different immunocompetent mice. **a**, MR alters fecal bacterial composition in *Apc*^*min+/−*^ mice. *Apc*^*min+/−*^ mice were fed with a CTRL diet or a MR diet (n = 12 mice on CTRL diet and 11 mice on MR diet, two-tailed unpaired Student’s t-test). The ratio of firmicutes/Bacteroidetes was calculated. **b**, MR alters the fecal abundance of *A. muciniphila*, Odoribacter, and Bifidobacterium. **c**, MR alters fecal bacterial composition in B6 mice. C57BL/6J (B6) donor mice were fed with a CTRL diet or a MR diet (n = 10 mice on CTRL diet and 10 mice on MR diet, two-tailed unpaired Student’s t-test). **d**, MR reduces firmicutes in the small intestine of B6 mice. C57BL/6J (B6) donor mice were fed with a CTRL diet or a MR diet. Total intestinal DNA samples were analyzed for microbiome using 16S rRNA gene amplicon sequencing as described in Methods (n = 10 mice/group, two-tailed unpaired Student’s t-test). **e**, MR reduces bacilli while increasing actinobacteria in the small intestine of B6 mice (n = 10 mice/group). Values are expressed as mean ± s.e.m. Details of statistical tests are included in the Methods. Source data

Bacterial 16S rRNA gene amplicon sequencing analyses revealed that MR profoundly impacted the gut microbiota in both tumour-bearing and tumour-free mice. In *Apc*^*min+/−*^ mice, MR significantly increased the Firmicutes/Bacteroidetes ratio (Extended Data Fig. 4a). Notably, MR depleted *Akkermansia muciniphila* and *Odoribacter* but increased *Bifidobacterium* in these tumour-bearing mice (Fig. 3a, Extended Data Fig. 4b and Supplementary Table 4). In tumour-free B6 mice, MR did not affect the Firmicutes/Bacteroidetes ratio (Extended Data Fig. 4c). However, it did significantly reduce faecal *Akkermansia* yet increased *Bifidobacterium* (Fig. 3b), as it did in *Apc*^*min+/−*^ mice. MR also substantially altered microbiota in the small intestines of B6 mice, including decrease of Firmicutes, primarily *Faecalibaculum* and *Lactobacillus*, and increase of *Bifidobacterium* (Fig. 3c and Extended Data Fig. 4d,e). *A. muciniphila*, *Odoribacter*, *Faecalibaculum*, *Lactobacillus* and *Bifidobacterium* are all major types of gut commensal bacteria frequently associated with colon carcinogenesis and therapeutic outcomes^21–24^. In particular, *A. muciniphila* is an abundant mucin-degrading intestinal bacterium^25,26^ that has many beneficial impacts on immunity, including an increase in the efficacy of anti-PD-1 immunotherapy^27^. Because the intestinal mucosal layer is an important nutrient-rich niche to harbour gut microbiota, we tested the possibility that MR may affect the above major commensal bacteria by modulating mucin production. Indeed, the expression levels of *Muc2*, which encodes a prominent member of mucin family secreted from goblet cells in the colonic epithelium to form the mucus gel layer^28^, and *Gal3st2*, which encodes a major galactose-3-*O*-sulfotransferase responsible for the synthesis of colonic sulphomucin^29^, were reduced in the colon tissue of methionine-restricted *Apc*^*min+/−*^ mice (Fig. 3d). The fraction of mucin-positive colonic epithelium was also significantly reduced in these mice (Fig. 3e).

**Fig. 4:**
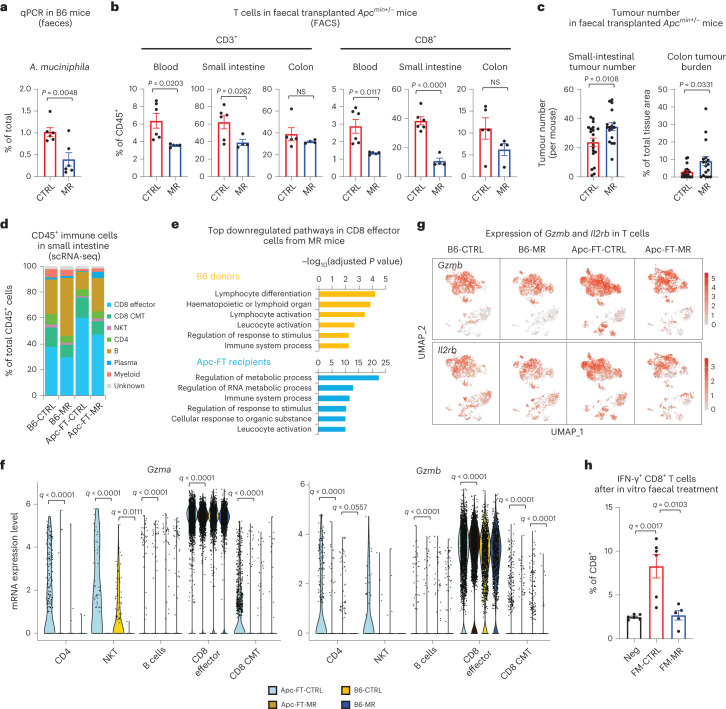
Methionine restriction promotes intestinal cancer progression and suppresses antitumour immunity through gut microbiota in immunocompetent mice. **a**, MR reduced *A. muciniphila* in B6 mice (*n* = 6 mice per group, two-tailed unpaired Student’s *t*-test). **b**, *Apc*^*min+/−*^ mice transplanted with faeces from MR diet-fed B6 mice had reduced abundance of CD3^+^ and CD8^+^ T cells in the blood and small intestine (*n* = 6 mice transplanted with faeces from mice fed CTRL diet and four to five mice transplanted with faeces from mice fed MR diet, two-tailed unpaired Student’s *t*-test). **c**, *Apc*^*min+/−*^ mice transplanted with faeces from MR diet-fed B6 mice had more intestinal tumours (*n* = 20 mice transplanted with faeces from mice fed a CTRL diet and 17 mice transplanted with faeces from mice fed an MR diet, two-tailed unpaired Student’s *t*-test). **d**, MR and MR diet-trained faecal microbiota reduced the fraction of CD8^+^ T cells in the small intestine. Total CD45^+^ immune cells from the small intestine of B6 donors and *Apc*^*min+/−*^ faecal recipient mice were analysed by scRNA-seq. **e**, CD8^+^ effector T cells from MR diet-fed B6 donor mice and *Apc*^*min+/−*^ faecal recipient mice had reduced expression of genes involved in immune cell function and activation in the small intestine. Significantly downregulated genes in CD8 effector cells in B6-MR mice and Apc-FT-MR mice in Supplementary Table 7 were analysed for enriched pathways (Methods). Top pathways identified in Gene Ontology, KEGG and Reactome are shown. **f**, Violin plots showing the reduced expression of *Gzma* and *Gzmb* in different groups of immune cells in B6-MR and Apc-FT-MR mice. **g**, Feature plots showing the reduced expression of *Ifngr1* and *Il2rb* in sub-T cell groups in B6-MR and Apc-FT-MR mice. **h**, Faecal microbiota from MR diet-fed mice failed to induce the expression of IFN-γ in CD8^+^ T cells. Purified mouse PBMCs were treated with faecal microbiota (FM) from CTRL diet or MR diet-fed donor mice for 12 h in vitro as described in Methods. The fraction of IFN-γ^+^CD8^+^ T cells was analysed by flow cytometry (*n* = 5 biological replicates per sample, Kruskal–Wallis test). Values are expressed as the mean ± s.e.m. Details of statistical tests are in Methods. NS, not significant. Source data

**Extended Data Fig. 5:**
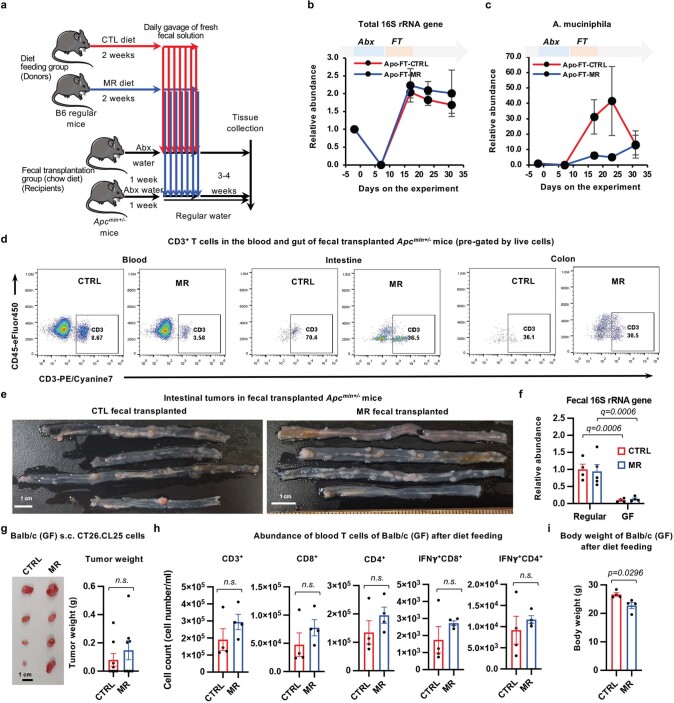
Dietary methionine restriction promotes intestinal cancer progression and suppresses anti-tumor immunity through gut microbiota in immunocompetent mice. **a**, Schematic of the fecal transplantation experiment. C57BL/6J mice were fed with either CTRL diet or a MR diet for 2 weeks. Fresh fecal solutions from these fed mice were then gavaged into antibiotic treated *Apc*^*min+/−*^ mice daily for one week. The recipient *Apc*^*min+/−*^ mice were fed with regular chow diet for the duration of the experiment, and intestinal tumors were analyzed 2-3 weeks after fecal transplantation. Mouse cartoon images were drawn by using pictures from Servier Medical Art (https://smart.servier.com/wp-content/uploads/2016/10/Animals.pptx). Servier Medical Art by Servier is licensed under a Creative Commons Attribution 3.0 Unported License (https://creativecommons.org/licenses/by/3.0/). **b-c**, Relative fecal abundance of total 16S rRNA gene and *A. muciniphila* in recipient *Apc*^*min+/−*^ mice during antibiotic depletion of gut microbiota and fecal transplantation. The recipient *Apc*^*min+/−*^ mice were treated with antibiotics (Abx) and fecal transplanted (FT) as described in Methods. Their feces were collected at indicated days to confirm the success of gut bacteria depletion and fecal transplantation by qPCR (n = 5 FT-CTRL and 7 FT-MR). **d**, *Apc*^*min+/−*^ mice transplanted with feces from MR diet fed C57BL/6J mice have reduced abundance of CD3^+^ T cells in the blood and small intestine. Representative flow cytometry plots are shown. **e**, *Apc*^*min+/−*^ mice transplanted with feces from MR diet-fed C57BL/6J mice have more intestinal tumors. Representative tumor images are shown. **f**, Fecal bacteria levels in regular and GF mice after CTRL or MR diet feeding (n = 4, 5, 4, and 4 mice/group, 2-way ANOVA). **g–h**, MR fails to significantly alters tumor growth and circulating T cell abundance in germ-free Balb/c mice. Germ-free Balb/c mice fed with irradiated CTRL or MR diets were subcutaneously injected with CT26.CL25 cells as described in Methods (n = 4 mice/8 tumor injections/group, two-tailed unpaired Student’s t-test). **i**, MR reduces body weight of germ-free Balb/c mice (n = 4 mice/group, two-tailed unpaired Student’s t-test). Values in are expressed as mean ± s.e.m. Details of statistical tests are included in the Methods. Source data

To assess the importance of the gut microbiota in mediating dietary methionine-induced modulation of T cell activation and tumour progression in the intestine, we transplanted the entire faecal microbial contents from tumour-free B6 mice on either MR or control diets to antibiotic-treated *Apc*^*min+/−*^ mice fed with chow diet (Extended Data Fig. 5a). Quantitative PCR (qPCR) analysis showed that the abundance of faecal *A. muciniphila* was significantly reduced in methionine-restricted B6 donor mice (Fig. 4a) and this trend was maintained at the early phase after faecal transplantation in the recipient *Apc*^*min+/−*^ mice (Extended Data Fig. 5b,c). Remarkably, faecal transplantation from MR diet-fed tumour-free B6 mice was sufficient to induce a dramatic reduction of the fraction of CD3^+^ and CD8^+^ T cells in the blood and small intestine (Fig. 4b and Extended Data Fig. 5d) and increase the tumour number and burden in the intestines in recipient *Apc*^*min+/−*^ mice (Fig. 4c and Extended Data Fig. 5e). Furthermore, MR pre-feeding did not significantly alter the growth of subcutaneously grafted CT26.CL25 tumours nor the abundance of circulating T cells in germ-free Balb/c mice (Extended Data Fig. 5f–h). However, interestingly, MR was still able to significantly reduce the body weight of these germ-free mice (Extended Data Fig. 5i), suggesting that the impact of MR on body weight is independent of microbiota. These observations support our hypothesis that the gut microbiota mediates, at least in part, the influence of dietary methionine on systemic immune function, antitumour immunity, and thus tumour progression.

**Extended Data Fig. 6:**
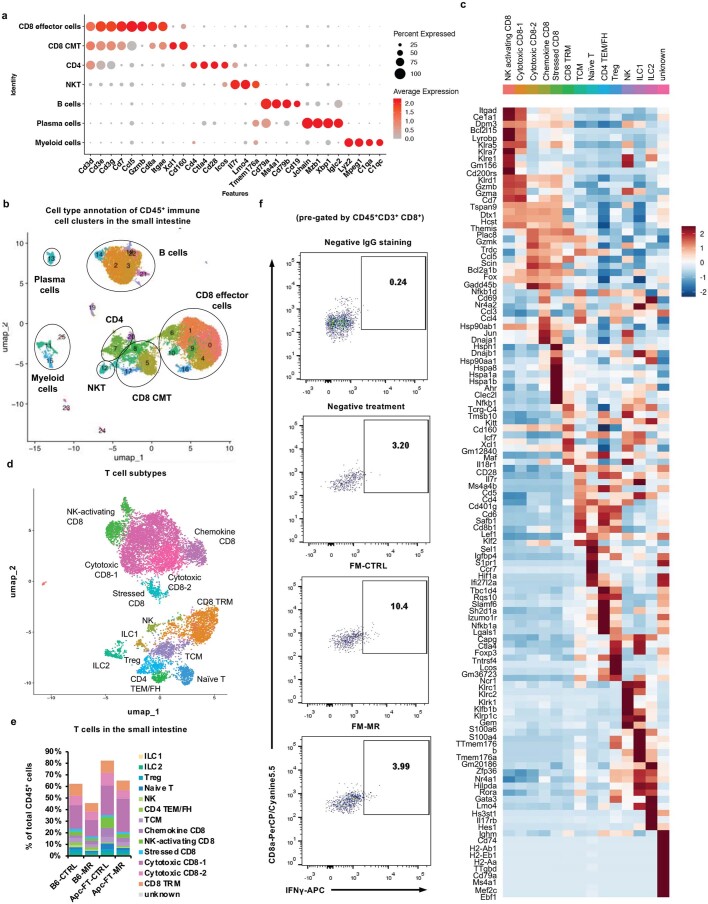
scRNA-seq analysis of CD45^+^ immune cells from the small intestine of diet-fed B6 donor or fecal transplanted *Apc*^*min+/−*^ mice. **a**, Dot plot showing the expression of marker genes in different function groups of total CD45^+^ immune cells. Cell population annotation of CD45^+^ immune cells from CTRL or MR fed B6 donor mice and from *Apc*^*min+/−*^ mice transplanted with fecal solution from CTRL or MR B6 mice. **b**, Total CD45^+^ immune cells from the whole small intestine of indicated mice were analyzed by scRNA-seq as described in Methods. **c**, Heat map showing the expression of marker genes in different sub-groups of T cells. **d**, Sub T cell group annotation. **e**, Dietary methionine restriction and MR diet-trained fecal microbiota reduces the fraction of several subgroups of intestinal CD8^+^ T cells. **f**, Representative FACS plots for in vitro activation of T cells by fecal microbes from control diet or MR diet fed B6 mice. Source data

Further analysis of total CD45^+^ immune cells in the small intestine of diet-fed tumour-free B6 donor mice (B6-CTRL and B6-MR) and chow-fed faecal recipient *Apc*^*min+/−*^ mice (Apc-FT-CTRL and Apc-FT-MR) by single-cell RNA-sequencing (scRNA-seq) showed that small-intestinal CD45^+^ immune cells could be categorized into seven functional groups (with 26 clusters) based on the expression patterns of known marker genes (Extended Data Fig. 6a,b and Supplementary Tables 5 and 6a). In line with the results in MR-fed *Apc*^*min+/−*^ mice (Fig. 4b), MR diet feeding reduced the total T cells, particularly CD8^+^ effector T cells and central memory T cells in B6 donor mice, but unexpectedly increased the total B cell abundance (Fig. 4d and Supplementary Table 6a). Notably, these MR diet-induced alterations of gut immune cells in B6 donor mice were transferred to the faecal recipient *Apc*^*min+/−*^ mice (Fig. 4d and Supplementary Table 6a), even though they were fed a regular chow diet. These findings indicate that the gut microbiota plays a major role in mediating the impacts of dietary methionine on gut immunity. Moreover, CD8^+^ effector T cells in both B6-MR and Apc-FT-MR mice had significant downregulation of genes in pathways mediating immune cell differentiation and activation compared to those in B6-CTRL and Apc-FT-CTRL mice, respectively (Fig. 4e and Supplementary Table 7). Different immune cells from B6-MR and Apc-FT-MR mice also had significantly reduced expression of several genes involved in cytotoxic activity and T cell-mediated immune responses (Supplementary Table 7). Particularly, their natural killer T cells had significantly reduced expression of *Gzma* and their CD4^+^ T and CD8^+^ central memory T cells had reduced expression of *Gzmb* (Fig. 4f and Supplementary Table 7). Additional subcategorizing of T cells (Extended Data Fig. 6c,d) showed that B6-MR and Apc-FT-MR mice had reduced abundance of central memory T cells, natural killer-activating CD8^+^ T cells, tissue-resident memory CD8^+^ T cells, and certain types of cytotoxic CD8^+^ T cells in their small intestines (Extended Data Fig. 6e and Supplementary Table 6b), many of which had reduced expression of *Gzmb* and *Il2rb* (Fig. 4g). Finally, while in vitro incubation of isolated mouse peripheral blood mononuclear cells (PBMCs) with faecal microorganisms from CTRL diet-fed B6 mice significantly increased the fraction of IFN-γ^+^CD8^+^ T cells, faecal microorganisms from MR diet-fed mice completely failed to do so (Fig. 4h and Extended Data Fig. 6f). Together, our findings strongly support the notion that dietary methionine exerts a large portion of its impacts on gut antitumour immunity through the gut microbiota.

## Methionine restriction reduces faecal hydrogen sulfide production and suppresses antitumour immunity

**Fig. 5:**
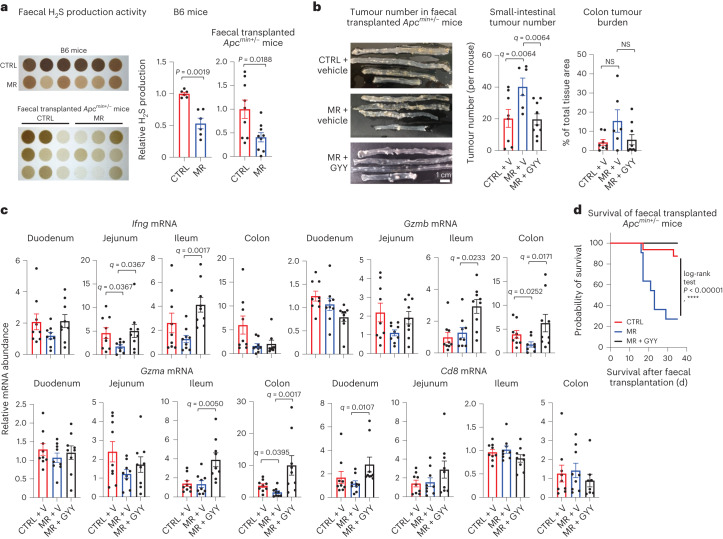
Methionine restriction reduces faecal hydrogen sulfide production and suppresses antitumour immunity in immunocompetent *Apc*^*min+/−*^ mice. **a**, MR reduced faecal H_2_S production activity. Faeces from CTRL or MR diet-fed C57BL/6J donor mice as well as respective *Apc*^*min+/−*^ recipient mice were subjected to H_2_S production assay using the lead sulfide assay as described in Methods (*n* = 6 mice each for C57BL/6J donor groups; 9 mice each for *Apc*^*min+/−*^ recipient groups, two-tailed unpaired Student’s *t*-test). **b**, Oral supplementation of GYY4137 rescues MR-induced increase of tumour progression in *Apc*^*min+/−*^ mice. *Apc*^*min+/−*^ mice transplanted with faeces from CTRL- or MR-fed B6 mice were oral gavaged daily with vehicle (V) or GYY4137 (GYY) as described in Methods (*n* = 8 mice on CTRL diet, 6 mice on MR diet and 9 mice on MR diet + GYY, Kruskal–Wallis test). **c**, Oral supplementation of GYY4137 rescued MR-induced reduction of *Ifng*, *Gzma* and *Gzmb* mRNA in the tumour-containing intestine of *Apc*^*min+/−*^ mice. *Apc*^*min+/−*^ mice were treated as in **b**, and the mRNA levels of indicated genes were analysed by qPCR (*n* = 9 mice per group, Kruskal–Wallis test). **d**, Oral supplementation of GYY4137 rescued the survival of *Apc*^*min+/−*^ mice on MR diet. *Apc*^*min+/−*^ mice were treated as in **b** (*n* = 16 mice on CTRL diet, 11 mice on MR diet and 9 mice on MR diet + GYY, log-rank test). Values are expressed as the mean ± s.e.m., except in **d**. Details of statistical tests are in Methods. NS, not significant. Source data

The gut microbiota interacts with the host immune system through multiple complex mechanisms^19^. Since gut microbial metabolites are the direct products of dietary interventions, we analysed faecal metabolites by metabolomics analysis. In B6 donor mice, MR significantly altered the abundance of 24 metabolites associated with fatty acid oxidation (Supplementary Table 8a and Supplementary Fig. 2). However, very few of these changes were sustained 3 weeks after transplantation into the recipient mice (Supplementary Table 8b). On the other hand, as expected from the restriction of a sulfur-containing amino acid, faecal hydrogen sulfide (H_2_S) production activity was significantly reduced in faeces from MR diet-fed B6 mice, and this reduction was preserved in MR faecal recipient *Apc*^*min+/−*^ mice 3 weeks after transplantation (Fig. 5a). H_2_S is a gasotransmitter with a wide range of implications in cancer, ageing and age-associated diseases^30^. It is also an endogenous potentiator of T cell activation^31^. The gut is the major H_2_S-producing organ, where the gut microbiota is responsible for about half of this production^32^. Therefore, dietary methionine might regulate T cell activation by supporting the H_2_S-producing activity of gut bacteria.

In line with this possibility, daily oral gavage of GYY4137, a water-soluble slow-release H_2_S donor^33^, significantly reduced the small-intestinal tumour number in *Apc*^*min+/−*^ mice transplanted with faeces from MR-fed B6 mice (Fig. 5b; MR + GYY), along with the rescue of the *Ifng*, *Gzma* and *Gzmb* mRNA levels in different segments of the intestine (Fig. 5c) and a striking rescue of their symptom-free survival (Fig. 5d). These observations strongly suggest that defective faecal H_2_S production is responsible, at least partially, for MR-induced deficiency of immune cell activation and tumour progression in *Apc*^*min+/−*^ mice.

**Fig. 6:**
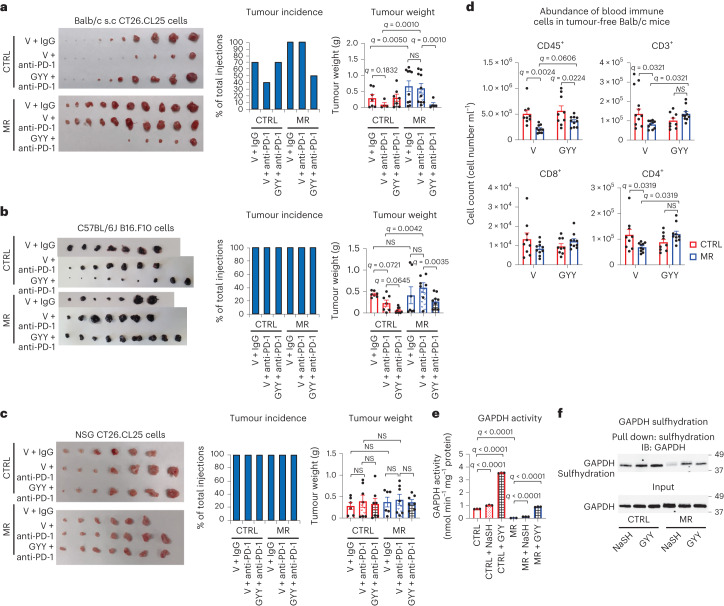
Supplementation of hydrogen sulfide donors enhances antitumour immunity in immunocompetent mice. **a**, Oral supplementation of GYY4137 rescued MR-induced resistance of subcutaneously grafted CT26.CL25 tumours to anti-PD-1 treatment in Balb/c mice (*n* = 10 mice/10 tumour injections per group, two-way ANOVA). **b**, Oral supplementation of GYY rescued MR-induced resistance of subcutaneously grafted B16.F10 tumours to anti-PD-1 treatment in C57BL/6J mice (*n* = 7, 7, 9, 7, 7 and 9 tumours per group, two-way ANOVA). **c**, Neither MR nor oral supplementation of GYY4137 affected the growth of allografted CT26.CL25 tumours in immunodeficient NSG mice (*n* = 6, 7, 7, 6, 7 and 7 tumours per group, two-way ANOVA). **d**, Oral supplementation of GYY4137 rescued MR-induced reduction of CD3^+^ and CD4^+^ T cells in regular Balb/c mice. CTRL or MR-fed Balb/c mice were daily gavaged with GYY for 20 d. The total cell number of indicated blood T cells was analysed by flow cytometry (*n* = 9, 10, 9 and 10 mice per group, two-way ANOVA). **e**, Supplementation of H_2_S donors increased the activity of GAPDH in activated human PBMCs. Human PMBCs activated by CD3/CD28 beads and IL-2 were cultured in control RPMI 1640 medium containing 100 μM methionine (CTRL) or a methionine-restricted medium containing 10 μM methionine (MR) with or without 500 μM NaSH or GYY overnight. The activity of GAPDH was measured as described in Methods (*n* = 3 biological repeats per group, two-way ANOVA). **f**, Supplementation of H_2_S donors increased the sulfhydration of GAPDH in activated human PBMCs. Human PMBCs were treated as previously and sulfhydration of GAPDH was measured as described in Methods. Representative immunoblots are shown. Values are expressed as the mean ± s.e.m. Details of statistical tests are included in Methods. Source data

**Extended Data Fig. 7:**
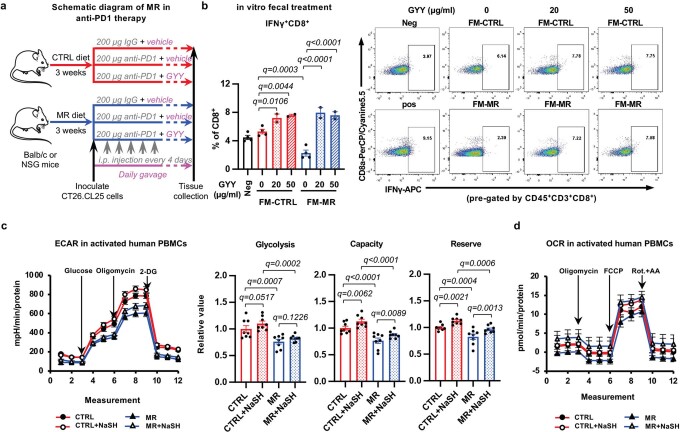
Hydrogen sulfide chemical donors rescue anti-tumor immunity in mice under dietary methionine restriction. **a**, Schematic of the GYY4137 and anti-PD1 immunotherapy experiment. Balb/c or NSG mice were fed with either CTRL diet or a MR diet for 3 weeks. After s.c. injected with 2 × 10^5^ CT26.CL25 cells, mice in each group were randomly divided with 3 groups to be daily gavaged with GYY4137, and i.p. injected with 200 µg control IgG or anti-PD-1 antibody every 4 days. Allografted tumors were monitored and analyzed 2-3 weeks after inoculation. Mouse cartoon images were drawn by using pictures from Servier Medical Art (https://smart.servier.com/wp-content/uploads/2016/10/Animals.pptx). Servier Medical Art by Servier is licensed under a Creative Commons Attribution 3.0 Unported License (https://creativecommons.org/licenses/by/3.0/). **b**, GYY4137 supplementation rescues the expression of IFNγ in CD8^+^ T cells treated with fecal microbiota from MR diet fed mice. Purified mouse PBMCs were treated with fecal microbiota (FM) from CTRL diet or MR diet fed donor mice plus indicated amount of GYY4137 for 6 hours in vitro as described in Methods. The fraction of IFNγ^+^ CD8^+^ T cells were analyzed by flow cytometry (n = 4 or 2 biological replicates/sample, 2-way ANOVA). **c**, Glycolysis of activated human PBMCs treated with indicated conditions. Cells were treated and ECAR in each group was measured as described in Methods (n = 8 biological replicates/group, 2-way ANOVA). **d**, Oxygen consumption of activated human PBMCs treated with indicated conditions. Cells were treated and OCR in each group was measured as described in Methods (n = 3 biological replicates/group). Values are expressed as mean ± s.e.m. Details of statistical tests are included in the Methods. Source data

When tested for its ability to rescue MR-induced resistance to anti-PD-1 immunotherapy (Extended Data Fig. 7a), GYY4137 sensitized subcutaneously grafted CT26.CL25 tumours in Balb/c mice fed with the MR diet to the anti-PD-1 treatment (Fig. 6a). GYY4137 also reduced weights of subcutaneously grafted B16.F10 melanoma tumours following anti-PD-1 treatment in C57BL/6J mice, although it did not impact the tumour incidence (Fig. 6b). This effect was completely absent in subcutaneously grafted CT26.CL25 tumours on immunodeficient NSG mice (Fig. 6c), indicating that dietary methionine and GYY modulate tumour growth through antitumour immunity. Consistent with these findings, daily oral gavage of GYY4137 significantly rescued the decrease of blood T cell counts induced by MR in Balb/c mice (Fig. 6d).

When tested in vitro, GYY4137 supplementation in culture medium significantly increased the IFN-γ^+^CD8^+^ T fraction in mouse PBMCs treated with faecal microorganisms from MR-fed B6 mice (Extended Data Fig. 7b), suggesting that MR diet-induced reduction of faecal H_2_S production directly contributes to the defective antitumour immunity. To further understand how H_2_S may promote immune cell abundance/activity, we investigated whether H_2_S donors can activate glycolysis of activated immune cells cultured with different levels of methionine, as H_2_S-mediated protein cysteine sulfhydration has been reported to augment GAPDH activity^34^. Consistent with this notion, overnight culture of activated human PBMCs in an MR medium substantially reduced the activity and sulfhydration of GAPDH (Fig. 6e,f; MR versus CTRL). Supplementation of NaSH, another potent H_2_S donor, or GYY, significantly enhanced the activity and sulfhydration of GAPDH in cells cultured in the CTRL medium and rescued the activity and sulfhydration of GAPDH in cells cultured in the MR medium (Fig. 6e,f). Additional Seahorse analyses showed that MR blunted the glycolytic activities but not oxygen consumption in activated human PBMCs (Extended Data Fig. 7c,d). Supplementation of NaSH enhanced the glycolytic capacity and glycolytic reserve in these cells (Extended Data Fig. 7c). Therefore, H_2_S may enhance the survival/activity of immune cells by increasing cysteine sulfhydration and glycolysis.

## Deficiency in gut microbiota-mediated hydrogen sulfide production impairs antitumour immunity

**Fig. 7:**
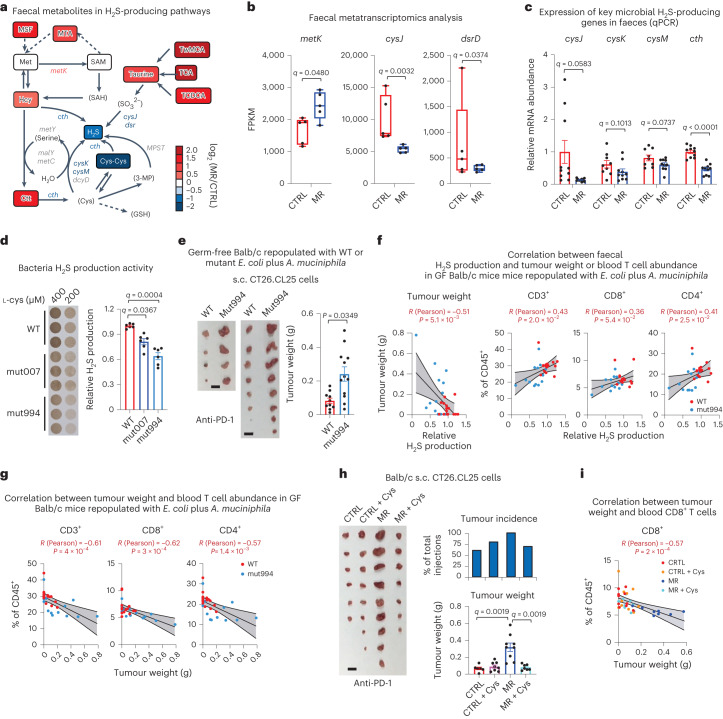
Deficiency in gut microbiota-mediated H_2_S production impairs antitumour immunity. **a**, MR altered faecal metabolites in H_2_S-producing pathways. Faecal metabolites were analysed by metabolomics, and the log ratios of the relative abundance of metabolites in MR/CTRL faeces were presented by a colour scale (*n* = 10 mice per group). **b**, MR altered the expression of key H_2_S-producing microbial genes in the faeces (*n* = 5 mice per group; box-and-whiskers plot, whiskers represent the minimum to maximum values, corrected using the Benjamini–Hochberg method for the false discovery rate). **c**, MR reduced the expression of indicated faecal microbial H_2_S-producing enzymes (qPCR using total bacterial 16S rRNA gene as a control, *n* = 10 mice per group, from an independent cohort, multiple two-tailed unpaired Student’s *t*-tests; one outlier in CTRL group was removed for *cysK* and *cysM* by >Q3 + 3.0 times the IQR). **d**, DecR mutant *E. coli* has reduced H_2_S-producing activity in vitro (*n* = 6 replicates, Kruskal–Wallis test). **e**, CT26.CL25 tumours exhibit increased growth in germ-free Balb/c mice repopulated with decR mutant *E. coli*/*A. muciniphila* after treatment with anti-PD-1 (*n* = 10 tumours for WT and 12 tumours for decR mutant, two-tailed unpaired Student’s *t*-test). Scale bars, 1 cm. **f**, The faecal H_2_S-producing activity was negatively correlated with the tumour weight yet positively correlated with the abundance of circulating T cells in germ-free Balb/c mice repopulated with *E. coli*/*A. muciniphila*. All mice in **e** were analysed (*n* = 15 mice for WT and 14 mice for decR mutant; two tailed, 95% confidence intervals are labelled). **g**, The tumour weight was negatively correlated with the abundance of circulating T cells in germ-free Balb/c mice repopulated with *E. coli/A. muciniphila* (*n* = 15 mice for WT and 14 mice for decR mutant; two tailed, 95% confidence intervals are labelled). **h**, Dietary cysteine supplementation rescued MR-induced growth and resistance of subcutaneous (s.c.) CT26.CL25 tumours to anti-PD-1 treatment in Balb/c mice (*n* = 10 mice/10 tumour injections per group, two-way ANOVA). Scale bar, 1 cm. **i**, The tumour weight was negatively correlated with the abundance of circulating CD8^+^ T cells in Balb/c mice fed with indicated diets (*n* = 10 mice per group; two tailed, 95% confidence intervals are labelled). Values are expressed as the mean ± s.e.m., except in **b**, **f**, **g** and **i**. Details of statistical tests are in Methods. Ctt, cystathionine; Hcy, homocysteine. Source data

**Extended Data Fig. 8:**
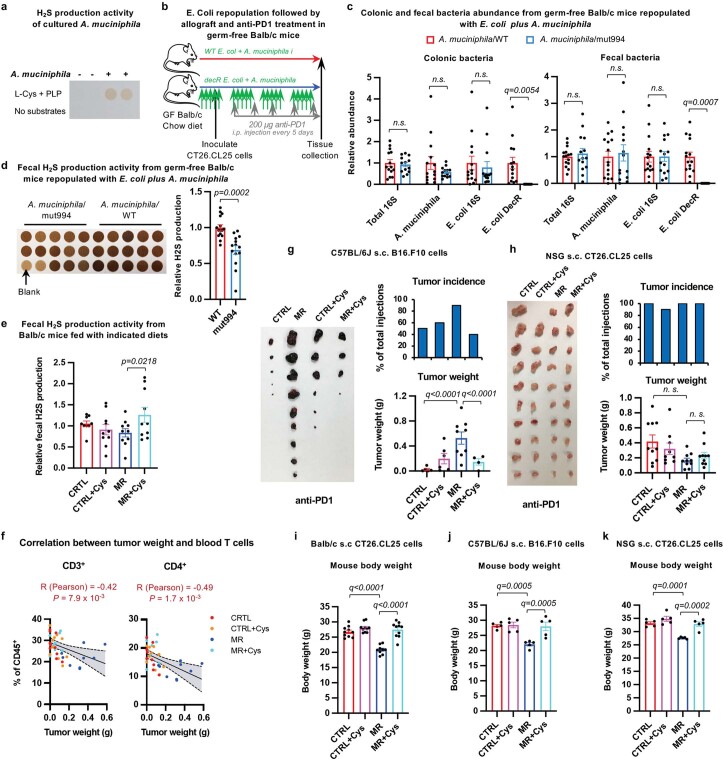
Dietary methionine restriction impairs gut microbiota-mediated H_2_S production from cysteine. **a**, *A. muciniphila* produces H_2_S in vitro. **b**, Schematic of anti-PD-1 immunotherapy in mice repopulated with WT or decR mutant *E. coli* plus *A. muciniphila*. Germ free (GF) Balb/c mice were gavaged with WT or decR mutant *E. coli* plus *A. muciniphila* 5 days a week for 4 weeks. Five days after the first bacteria gavage, 2 × 10^5^ CT26.CL25 cells were s.c. injected, immediately followed with i.p. injection of 200 µg/injection anti-PD-1 antibody every 5 days. Mouse cartoon images were drawn by using pictures from Servier Medical Art (https://smart.servier.com/wp-content/uploads/2016/10/Animals.pptx). Servier Medical Art by Servier is licensed under a Creative Commons Attribution 3.0 Unported License (https://creativecommons.org/licenses/by/3.0/). **c**, The relative abundance of indicated bacteria in the colons and feces of mice in (**b**) (n = 15 mice for WT and 14 mice for decR mutant, multiple two-tailed unpaired Student’s t-test). **d**, Fecal H_2_S producing activities of mice in (**b**) (n = 15 mice for WT and 14 mice for decR mutant, two-tailed unpaired Student’s t-test). 400 µM of cysteine was used as a substrate. **e**, Dietary cysteine supplementation in MR diet increases fecal H_2_S production. 10 mM of cysteine was used as a substrate (n = 10 mice/group, 2-way ANOVA; one outlier in CTRL group was removed by <Q1-3.0*IQR). **f**, The tumor weight is negatively correlated with the abundance of circulating T cells in Balb/c mice (n = 10 mice/group). **g**, Dietary cysteine supplementation does not impact the growth and the anti-PD1 response of CT26.CL25 tumors in NSG mice (n = 5 mice/10 tumor injections/group, 2-way ANOVA). **h**, Dietary cysteine supplementation rescues MR-induced growth and resistance of B16.F10 tumors to anti-PD1 treatment in C57BL/6J mice (n = 5 mice/10 tumor injections/group, 2-way ANOVA). **i**, Dietary cysteine supplementation rescues MR-induced body weight loss in Balb/c mice (n = 10 mice/group, 2-way ANOVA). **j–k**, Dietary cysteine supplementation rescues MR-induced body weight loss in C57BL/6J and NSG mice (n = 5 mice/group, 2-way ANOVA). Values are expressed as mean ± s.e.m. Details of statistical tests are included in the Methods. Source data

To better understand how MR reduces H_2_S production from gut microorganisms, we reanalysed faecal metabolites using a different untargeted metabolomic platform in an independent cohort of CTRL or MR diet-fed B6 mice (Supplementary Table 8c). Intriguingly but not surprisingly, alliin, a natural sulfoxide derived from l-cysteine, and l-cystine, the oxidation dimer of l-cysteine, were among the most dramatically reduced metabolites from MR diet-fed faeces (Supplementary Table 8c), indicating that one of the most significantly altered pathways in faeces from MR fed mice is the sulfur amino acid metabolism pathway. Further analysis of metabolites in the sulfur amino acid metabolic pathways showed that faeces from MR-fed mice accumulated several sulfur-containing metabolites, particularly cystathionine, homocysteine and taurine and taurine-conjugated bile acids, but were depleted of l-cystine (Cys-Cys; Fig. 7a), suggesting that reduced H_2_S production is possibly due to diminished conversion of the above sulfur-containing metabolites into cysteine and/or H_2_S. In support of this possibility, metatranscriptomic analysis and subsequent qPCR validation showed that faecal bacteria from MR-fed mice exhibited reduction of the RNA levels of *cysJ* and *dsrD*, which encode two enzymes involved in H_2_S production from sulfite and taurine, *cysK* and *cysM*, which encode two enzymes converting cysteine to H_2_S, and *cth*, which encodes an enzyme involved in cysteine and H_2_S production from homocysteine and cystathionine^35^ (Fig. 7a–c). Furthermore, many faecal bacterial species from our MR or CRTL-fed mice expressed various H_2_S-producing enzymes^35^ (Supplementary Table 9). For instance, *A. muciniphila*, one of the most significantly downregulated commensal gut bacterial species in the faeces of different strains of MR-fed mice (Fig. 3a,b), had detectable transcripts of *cysK*, *metY* and *malY*, genes that encode three key H_2_S-producing enzymes (Supplementary Table 9) and displayed an appreciable H_2_S producing activity when measured in vitro (Extended Data Fig. 8a). *Bifidobacterium globosum*, a member of the *Bifidobacterium* genus, which was one of the most significantly up-regulated taxonomic groups in both faeces and small intestine of MR-fed mice (Fig. 3a–c), also actively expressed *metY* and *malY* (Supplementary Table 9).

Next, we sought to test whether MR-resulted suppression of gut microbial H_2_S production directly contributes to enhanced tumour growth and impaired antitumour immunity observed in MR-fed mice. We performed a proof-of-concept experiment by repopulating germ-free Balb/c mice fed on the chow diet with WT *Escherichia coli* or a mutant *E. coli* with deletion of decR, a transcriptional factor controlling detoxification of l-cysteine and production of H_2_S in *E. coli*^36^. *E. coli* decR mutants with defective H_2_S production have been previously used to elucidate the role of microbial H_2_S in chronic kidney disease^37^. However, mono-colonization of neither WT nor decR mutant *E. coli* into germ-free Balb/c mice significantly impacted the tumour growth of mice subcutaneously inoculated with CT26.CL25 cells (Supplementary Fig. 3). Since many faecal bacterial species in our experimental mice expressed H_2_S-producing enzymes (Supplementary Table 9), thus possibly having a high background H_2_S production, we chose to co-colonize WT or decR mutant *E. coli* with *A. muciniphila* (Extended Data Fig. 8b). A major gut bacterial species sensitive to MR in different mouse strains (Fig. 3a,b) and a weaker H_2_S producer (Extended Data Fig. 8a), *A.* *muciniphila* is one of known keystone species of human microbiome^38,39^ important in the regulation of the efficacy of anti-PD-1 immunotherapy^27^. The repopulated germ-free Balb/c mice were then subcutaneously inoculated with CT26.CL25 cells and treated with anti-PD-1 (Extended Data Fig. 8b). We confirmed that *E. coli* decR mutants displayed an expected defect in H_2_S production in vitro (Fig. 7d) and 3 weeks after repopulation into germ-free Balb/c mice (Extended Data Fig. 8c,d). Remarkably, subcutaneously grafted CT26.CL25 tumours were significantly bigger in germ-free Balb/c mice repopulated with decR mutant *E. coli* plus *A. muciniphila* than those in germ-free Balb/c mice repopulated with WT *E. coli* plus *A. muciniphila* after anti-PD-1 treatment (Fig. 7e). Importantly, the faecal H_2_S production activity was negatively correlated with the tumour weight yet positively correlated with the fraction of circulating T cells (Fig. 7f), and there was also significant negative correlation between the tumour weight and the abundance of circulating T cells (Fig. 7g). Together, these observations indicate that reduced H_2_S production from gut microbiota directly impairs antitumour immunity and dampens the response of allograft tumours to antitumour immunotherapy.

Finally, we tested whether supplementation of cysteine, a major precursor of H_2_S (Fig. 7a), could rescue MR-induced impairment in antitumour immunity and increase in tumour growth. Dietary supplementation of cysteine significantly increased faecal H_2_S production activity in Balb/c mice fed with the MR diet (Extended Data Fig. 8e) and rescued MR-induced resistance of subcutaneously grafted CT26.CL25 tumours to anti-PD-1 treatment (Fig. 7h). Additionally, the tumour weight in all experimental mice was significantly negatively correlated with the fraction of circulating T cells (Fig. 7i and Extended Data Fig. 8f). Dietary cysteine supplementation also rescued the MR-induced enhancement of growth of B16.F10 tumours and their resistance to anti-PD-1 treatment in immunocompetent B6 mice (Extended Data Fig. 8g). However, it failed to impact the growth of CT26.CL25 tumours in immunocompromised NSG mice in response to anti-PD-1 treatment (Extended Data Fig. 8h). Again, dietary cysteine supplementation was able to rescue MR-induced body weight loss in all mice regardless of their status of immunity (Extended Data Fig. 8i–k), indicating that dietary methionine and cysteine modulate antitumour immunity independently of their effects on body weight. Altogether, our data indicate that MR-induced reduction of microbial H_2_S production contributes to the observed suppression of antitumour immunity.

## Dietary methionine supplementation enhances antitumour immunity and suppresses tumour growth in immunocompetent mice

**Fig. 8:**
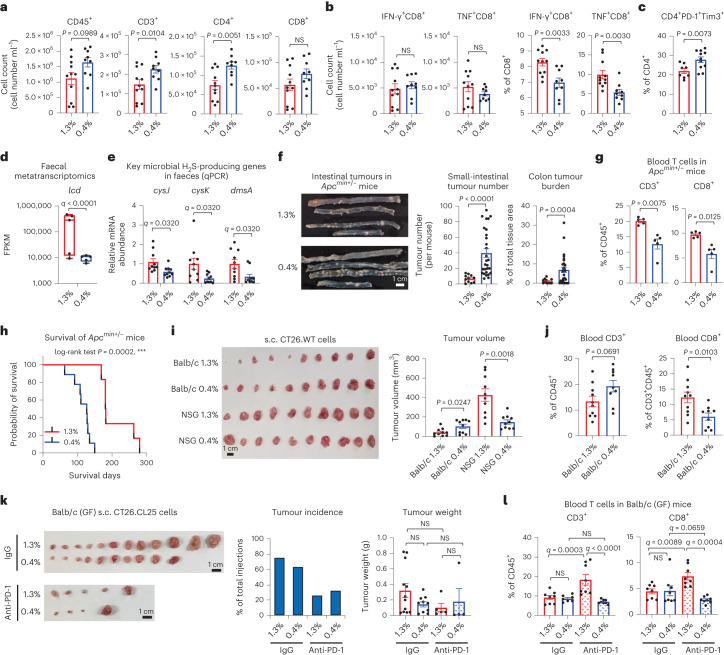
Dietary methionine supplementation activates T cells and suppresses tumour progression in immunocompetent mice. **a**, Dietary methionine supplementation reduced the abundance of circulating T cells (*n* = 11 mice on 1.3% diet and 9 mice on 0.4% diet, two-tailed unpaired Student’s *t*-test). **b**, Dietary methionine supplementation increased the abundance and fraction of activated CD8^+^ T cells (*n* = 11 mice on 1.3% diet and 9 mice on 0.4% diet, two-tailed unpaired Student’s *t*-test). **c**, Dietary methionine supplementation reduced exhaustion of circulating CD4^+^ T cells in C57BL/6J mice (*n* = 11 mice on 1.3% diet and 9 mice on 0.4% diet, two-tailed unpaired Student’s *t*-test). **d**, Dietary methionine supplementation increases the expression of microbial l-cysteine desulfhydrase (*lcd*) gene in faeces (*n* = 5 mice per group, box-and-whiskers plot, whiskers indicate minimum to maximum values, corrected using the Benjamini–Hochberg method for the false discovery rate). **e**, Dietary methionine supplementation increased the expression of key sulfur metabolic genes in faeces (*n* = 10 mice per group, from an independent experimental cohort, multiple Student’s *t*-tests). **f**, Dietary methionine supplementation inhibited tumour growth in *Apc*^*min+/−*^ mice (*n* = 11 mice on 1.3% diet and 28 mice on 0.4% diet, two-tailed unpaired Student’s *t*-test). **g**, Dietary methionine supplementation increased blood T cell fractions in *Apc*^*min+/−*^ mice (*n* = 5 mice per group, two-tailed unpaired Student’s *t*-test). **h**, Dietary methionine supplementation increased survival of *Apc*^*min+/−*^ mice (*n* = 9 mice on a 0.4% diet and 6 mice on a 1.3% diet. Log-rank test). **i**, Dietary methionine supplementation repressed tumour growth in immunocompetent mice but enhanced tumour growth in immunodeficient mice (*n* = 10 tumours per group, two-tailed unpaired Student’s *t*-test between 1.3% versus 0.4% only). **j**, Dietary methionine supplementation increased blood CD8^+^CD3^+^ T cells in Balb/c mice (*n* = 10 mice on a 1.3% diet and 9 mice on a 0.4% diet, two-tailed unpaired Student’s *t*-test). **k**, Dietary methionine supplementation dose not significantly affected tumour growth and response to anti-PD-1 treatment in germ-free Balb/c mice (*n* = 8 mice/16 tumour injections per group, two-way ANOVA; one outlier in the 1.3% + anti-PD-1 group was removed). **l**, The impact of dietary methionine supplementation on blood T cells in germ-free Balb/c mice (*n* = 8 mice per group, two-way ANOVA). Values are expressed as the mean ± s.e.m., except in **h**. Details of statistical tests are included in Methods. Source data

**Extended Data Fig. 9:**
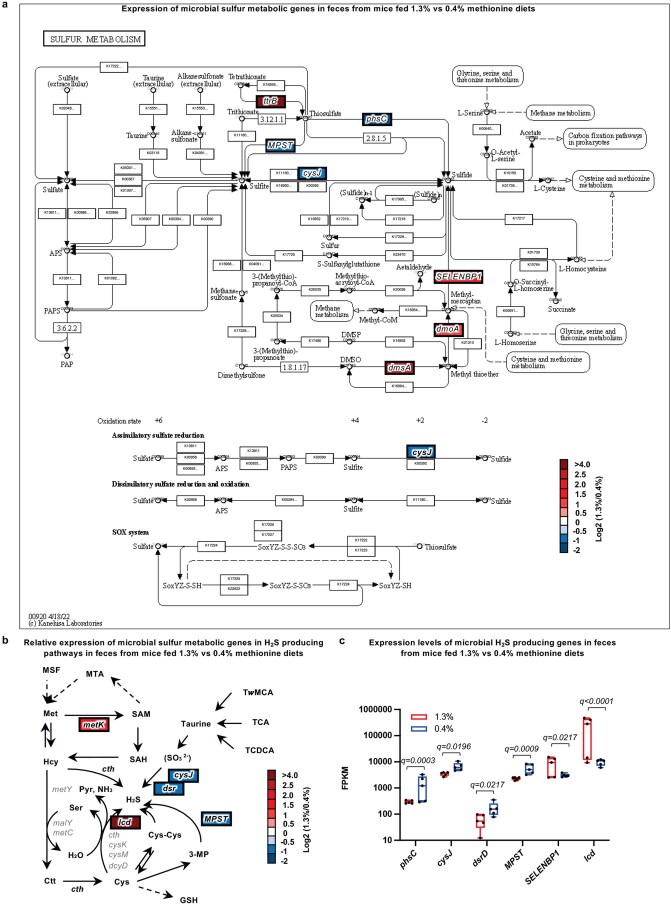
Dietary methionine supplementation alters microbial sulfur metabolic genes in feces of C57BL/6J mice. **a-b**, Dietary methionine supplementation alters the expression of several key microbial sulfur metabolic genes in the feces. Fecal RNAs from mice fed 1.3% or 0.4% methionine diets were subjected to metatranscriptomic analysis as described in Methods (n = 5 mice/group, all highlighted genes were significantly altered). The log ratios of the relative levels of indicated microbial gene in feces from mice fed 1.3% vs 0.4% methionine diets were presented by a color scale in a KEGG sulfur metabolism map (adapted from KEGG PATHWAY, https://www.genome.jp/entry/pathway+map00920) (**a**), or in an organic sulfur metabolic network (**b**). **c**, Dietary methionine supplementation dramatically increases the expression of microbial *lcd* gene in feces (n = 5 mice/group, Box and whiskers plot, whiskers: Min to Max). Multiple comparisons were corrected using the Benjamini-Hochberg method for the false-discovery rate. Source data

We further investigated whether dietary methionine supplementation could boost antitumour immunity. Intriguingly, compared to the chow diet containing 0.4% l-methionine, a custom-made methionine-supplemented chow diet containing 1.3% l-methionine reduced the abundance of circulating T cells, particularly CD4^+^ T cells while not significantly impacting CD8^+^ T cells in regular B6 mice (Fig. 8a and Supplementary Fig. 4a). These alterations were accompanied with an increased fraction of circulating IFN-γ^+^CD8^+^ and tumour necrosis factor (TNF)-positive CD8^+^ T cells (Fig. 8b and Supplementary Fig. 4b) and a reduced fraction of PD-1^+^Tim3^+^CD4^+^ T cells (Fig. 8c and Supplementary Fig. 4c). Furthermore, the expression of various faecal microbial sulfur metabolic genes was altered in these methionine diet-fed B6 mice (Extended Data Fig. 9a, shown on the KEGG sulfur metabolism map^40^, and Extended Data Fig. 9b). Particularly, the mRNA level of a highly expressed microbial enzyme mediating H_2_S production from cysteine, l-cysteine desulfhydrase, was dramatically induced by dietary methionine supplementation in faeces of B6 mice (Fig. 8d and Extended Data Fig. 9b,c). Additional qPCR analysis revealed that several other key sulfur metabolic genes involved in H_2_S production, such as *cysJ*, *cysK* and *dmsA*, were also significantly induced in faeces from an independent cohort of mice fed the methionine-supplemented diet (Fig. 8e).

In *Apc*^*min+/−*^ mice, dietary methionine supplementation reduced the intestinal tumour number and burden (Fig. 8f), increased the fractions of circulating CD3^+^ and CD8^+^ T cells (Fig. 8g and Supplementary Fig. 5a) and extended their healthspan (Fig. 8h). Remarkably, in the syngeneic CT26 tumour model, dietary methionine supplementation suppressed the growth of subcutaneously grafted CT26.WT tumours in immunocompetent Balb/c mice, yet it markedly enhanced their growth in immunocompromised NSG mice (Fig. 8i), further supporting the notion that the impact of dietary methionine on tumour progression is dependent on the status of the immune system. The suppression of tumour growth in Balb/c mice fed a high-methionine diet was coupled with a significantly increased fraction of circulating CD8^+^ T cells in these tumour-bearing mice (Fig. 8j and Supplementary Fig. 5b). Importantly, the tumour suppression and immune activation effects of dietary methionine supplementation in immunocompetent Balb/c mice were dependent on the gut microbiota, as this dietary intervention failed to suppress the growth of subcutaneously grafted CT26.CL25 tumours (Fig. 8k; IgG), and did not increase circulating CD8^+^ T cells (Fig. 8l, IgG; Supplementary Fig. 5c) in germ-free Balb/c mice. However, interestingly, anti-PD-1 treatment reduced the tumour incidence regardless of diets (Fig. 8k) and significantly increased the abundance of circulating CD3^+^ and CD8^+^ T cells (Fig. 8l) when germ-free Balb/c mice were fed with a 1.3% diet but not a 0.4% diet, indicating that in the presence of sufficient methionine, germ-free mice still possess a certain degree of antitumour immunity that can be boosted by anti-PD-1 antibody. In sum, our findings indicate that dietary methionine supplementation enhances antitumour immunity in immunocompetent mice partially through gut microbiota.

## Discussion

As a sulfur-containing proteinogenic amino acid, methionine has a broad array of cell-autonomous impacts on protein synthesis, histone methylation and redox homeostasis in both cancer cells and immune cells and, thereby, is essential for their proliferation, stress resistance and overall functions^2,3,10–12,41^. The net effect of dietary methionine on cancer growth, therefore, depends on the relative dependence of cancer cells versus immune cells on this nutrient in the tumour microenvironment. In the present study, we demonstrate that in addition to the reported cell-autonomous epigenetic alteration through SAM production in immune cells^12,13^, the impact of dietary methionine on tumour growth and antitumour immunity is also dependent, at least in part, on gut microbiota-mediated non-cell-autonomous activation of immune cells. Specifically, our findings support the notion that the restricted sulfur intake largely underlies MR-induced impairment of antitumour immunity, as dietary supplementation of a H_2_S donor or l-cysteine, a sulfur amino acid acting downstream of SAM, rescued dietary MR-induced resistance to anti-PD-1 treatment in immunocompetent mice but not in immunodeficient mice.

It is worth noting that in immunocompetent mice fed with the CTRL diet, oral supplementation of GYY or cysteine had a trend to increase tumour incidence/growth, especially after anti-PD-1 treatment (Figs. 6a and 7h and Extended Data Fig. 8g), suggesting that too much H_2_S production is detrimental to antitumour immunity. Therefore, a balanced level of H_2_S is required for a healthy gut immunity. Notably, in immunocompetent animals, MR has been reported to reduce tumour formation when this dietary intervention was started close to the initiation of tumourigenesis^42,43^. This observation is consistent with our finding that dietary MR will take 3–4 weeks to substantially affect circulating immune cell profiles and further suggests that the relative dependence of cancer cells versus immune cells on methionine in the tumour microenvironment may vary at different stages of tumour development.

Our study has several important clinical and translational implications. Firstly, due to its documented beneficial impacts on protection against metabolic diseases and ageing^4,5^ and repression of tumours^3,6–10,43^, dietary MR has been promoted as an effective dietary regimen to control these pathological conditions. However, our discovery in this study reveals an unexpected/neglected negative effect of MR on antitumour immunity, which suggests that in the clinical setting, MR may be more suitable to treat immunocompromised individuals with cancer. For immunocompetent individuals, there might be a therapeutic window before MR becomes detrimental to their antitumour immunity. Conversely, dietary methionine supplementation may boost antitumour immunity in immunocompetent individuals but will support tumour growth in immunocompromised individuals with cancer. This distinction may be critical for the success of clinical dietary interventions in cancer. In addition, the inverse relationship between the dietary protein intake and cancer risk that we observed in humans (Supplementary Table 1) concurs with our findings in mice. This supports the translational significance of our study that the low dietary methionine may favor cancer development/progress (while the high methionine might be protective) in both mice and humans.

Secondly, the discovery that the gut microbiota is involved in mediating the influence of MR on antitumour immunity and tumour progression implies that manipulating the microbiome could be applied for development of new therapeutic anticancer approaches in conjunction with dietary interventions.

Finally, the positive association between dietary methionine, the H_2_S-producing activity of gut microbiota, redox homeostasis in immune cells and antitumour immunity revealed in our study raises an exciting possibility that H_2_S chemical donors, when used at appropriate doses, may help to boost antitumour immunity and/or the efficacy of antitumour immunotherapies without direct impact on growth of tumour cells.

## Methods

### Cells

CT26.WT mouse colon cancer cells (American Type Culture Collection (ATCC) CRL-2638), CT26.CL25 cells (ATCC CRL-2639), a subclone of CT26.WT cells, and B16.F10 mouse melanoma cells (ATCC CRL-6475) were obtained from the ATCC. Human PBMCs were obtained from STEMCELL Technologies (70025). All cell lines were tested by the National Institute of Environmental Health Sciences (NIEHS) Quality Analysis Laboratory before inoculating into experimental mice.

### Animal experiments

All mice were housed in the animal facility at the NIEHS with an ambient temperature around 21–23 °C, humidity of 40–60%, and a dark–light cycle of 12 h. They were maintained under strict specific pathogen-free conditions. All mice were housed in micro-isolator static cages (Techniplast) with autoclaved nesting material (Nestlet, Ancare) and housed on hardwood bedding (Sani-chips, P.J. Murphy). All animals were housed, cared for and used in compliance with the Guide for the Care and Use of Laboratory Animals and housed and used in an Association for the Assessment and Accreditation of Laboratory Animal Care, International (AAALAC) Program.

An MR diet containing 0.172% dl-methionine without cystine (510029) and a control diet containing 0.86% dl-methionine without cystine (510027) were purchased from Dyets. The cysteine-supplemented CTRL diet containing 0.86% dl-methionine + 0.5% l-cysteine and MR diet containing 0.172% dl-methionine + 0.5% l-cysteine were custom made by Dyets. The custom-made 1.3% methionine-enriched chow diet and normal chow diet (National Institutes of Health (NIH)-31) were purchased from Harlan Teklad. All diets were tested by the NIEHS Quality Analysis Laboratory before feeding.

All animal procedures in this study were reviewed and approved by the NIEHS Animal Care and Use Committee under the Animal Study Proposal numbers ASP2014-0016 and ASP2017-0012. Both male and female mice were used in this study. We did not observe any significant sex differences in experiments using both sexes.

### Methionine diet feeding procedure in *Apc*^*min*+/−^ mice

Six- to eight-week-old *Apc*^*min+/−*^ mice (Jax: 002020, both sexes), with 8–12 mice per group, were fed with diets containing different methionine ad libitum for up to 8 weeks with free access to water. The first pair includes an MR diet containing 0.172% dl-methionine or a CTRL diet containing 0.86% dl-methionine, and the second pair includes the NIH-31 chow diet containing 0.4% methionine and a 1.3% methionine-supplemented chow diet. After 8 weeks of feeding, mice were euthanized by CO_2_ for tissue collection, and bled immediately afterward for other experiments.

To analyse the long-term impact of methionine diets on symptoms-free survival of *Apc*^*min+/−*^ mice, a small group of *Apc*^*min+/−*^ mice were maintained on their respective diets. Their health status was monitored twice a week, and mice were removed from the study (euthanized) when they lost 20% of their starting body weight, experienced severe rectal bleeding or displayed any signs of distress such as hunching, laboured breathing, incoordination, seizures, lethargy or becoming prostrate.

### Methionine diet feeding procedure in AOM-DSS CRC mice

Six- to eight-week-old C57BL/6J mice (Jax: 000664, females) were fed with CTRL (19 mice) or MR (20 mice) diets for 3 weeks. They were then intraperitoneally injected with 10 milligram per kilogram of body weight AOM. One week later, they were treated with 2% DSS in drinking water for 7 d, then regular water for the rest of the procedure. During the DSS treatment week and the following week, the body weight and health status of the experimental animals were monitored twice a day, and any mice experiencing more than 25% of their starting body weight, severe rectal bleeding, or displayed any signs of distress, such as hunching, laboured breathing, incoordination, seizures and lethargy, were euthanized. After this period, their health status was monitored once a week, and mice were euthanized when they lost 20% of their starting body weight, experienced any of above clinical signs or became prostrate. Colon tissues were dissected 14 weeks after the AOM injection to analyse the development and progression of CRC.

Another cohort of C57BL/6J male mice were fed with CTRL (24 mice) or MR (24 mice) diets for 4 weeks, then injected with 10 milligram per kilogram of body weight AOM, and treated with 2% DSS in drinking water for two cycles (7 d DSS water treatment and 14 d regular water break). Since most of mice in the MR group died or had to be removed from the experiment due to health concerns during the procedure, the experiment was terminated before reaching the endpoint.

### Orthotopic colon cancer model

Six- to eight-week-old BALB/c mice (Jax: 000651, males), with 10 mice per group, were fed with CTRL or MR diets for 3–4 weeks. They were then orthotopically (rectum) implanted with 1 × 10^4^ mouse colon cancer CT26.CL25 cells for each mouse. Mice were continually fed with their respective diets for additional 4–5 weeks.

### Allograft experiments and PD-1 treatment

Six- to eight-week-old BALB/c (Jax: 000651) and NSG (Jax: 005557) male mice, five to eight mice per group, were fed with two groups of high-methionine and low-methionine diets as described above for 3 weeks. Mice were then inoculated subcutaneously with 2 × 10^5^ mouse colon cancer CT26.WT cells and continually fed with the different methionine diets for an additional 3–4 weeks.

For the immunotherapy experiments with anti-PD-1, the mice were first fed with different methionine diets for 3 weeks, and then inoculated subcutaneously with 2 × 10^5^ mouse colon cancer CT26.CL25 cells. Isotype-matched control antibody rat IgG2a (BioXCell, BE0089) and rat anti-PD-1 (BioXCell, BE0146) were given intraperitoneally at a dose of 200 μg per mouse on day 4 after cell inoculation, then every 4 d for the duration of the experiment. Mice were continually fed with their respective diets for about additional 3–4 weeks with free access to water.

For all experimental mice in this procedure, their body weight and health status were monitored weekly during the first 3–4 weeks of diet feeding period. After cell inoculation, their body weight, tumour size and health status were monitored twice weekly. If an animal became hunched, lost more than 20% of its initial body weight or the tumour size exceeded 1.5 cm^3^, interfered with normal ambulation or ulcerated, the animal was removed and euthanized by CO_2_.

### Faecal transplantation procedure

One cohort of 6- to 8-week-old C57BL/6J male mice fed with control or MR diets (six mice/diet) for 2 weeks were used as donors to provide fresh faeces for faecal transplantation. Gut microbiota depletion and faecal transplantation were conducted as follows: 8- to 10-week-old *Apc*^*min+/−*^ mice (both sexes) fed on regular chow diet were given autoclaved water subjected to reverse osmosis or deionized water containing an antibiotic cocktail (1 g l^−1^ ampicillin and 0.575 g l^−1^ enrofloxacin) for 1 week and were then inoculated daily by oral gavage for 7 d with 100 μl per mouse of faecal solution prepared with combined faeces freshly collected from donor C57BL/6J mice (1:1 dilution of faeces with PBS). *Apc*^*min+/−*^ mice were maintained with sterile water subjected to reverse osmosis or deionized water and chow diet during faecal transplantation and afterward. They were monitored for their health status as described above daily, then analysed for intestinal tumour formation 3–4 weeks after the faecal transplantation.

### GYY4137 supplementation procedure

Six- to eight-week-old C57BL/6J, BALB/c mice and immunodeficient NSG mice, all males, with five to eight mice per group, were divided into six groups each, with three groups fed with the control diet and three groups fed with the MR diet for 3 weeks. Starting from day 21, mice in IgG group and one of the anti-PD-1 groups were dosed daily with a vehicle carboxymethyl cellulose, while mice in the second anti-PD-1 group were dosed daily with 50 milligram per kilogram of body weight GYY4137, respectively, through gavage. Also, on day 21, they were inoculated (subcutaneously) with 2 × 10^5^ cancer cells (B16.F10 or CT26.CL25) and administered with 200 μg IgG or anti-PD-1 per mouse on day 4 after cell inoculation, then every 4 d for the duration of the experiment. The mice were continually fed with their respective diets for about additional 4 weeks with free access to water. For the faecal transplantation experiment, mice in the CTRL group and one of the MR groups were gavaged daily with 100 μg water, while mice in the other MR group were gavaged daily with 50 mg per kilogram of body weight GYY4137 during faecal transplantation and until the end of experiment.

### Wild-type or decR mutant *E. coli* repopulation, followed by allograft and anti-PD-1 treatment

Six- to eight-week-old germ-free Balb/c mice (Taconic, Balb/cAnNTac (GF), males), with four to eight mice per group, were gavaged with WT (Horizon discovery, OEC5042) or decR mutant (mut994, mutant (Horizon Discovery, OEC4987-200825994) *E. coli* (0.1 OD_600_/mouse) plus *A. muciniphila* (ATCC, BAA-835, 1 OD_600_/mouse) in 150 μl PBS 5 d a week for 4 weeks. Five days after the first gavage dosing, 2 × 10^5^ mouse colon cancer CT26.CL25 cells were subcutaneously inoculated, and 200 μg of anti-PD-1 was injected every 5 d. Faeces were collected for faecal H_2_S production assay 3 weeks after tumour cell inoculation using the lead acetate method with 400 μM cysteine as a substrate (please refer to ‘Lead sulfide assay for faecal H2S production’ for more details). Tumours were collected 4 weeks after tumour cell inoculation. To confirm the success of bacterial colonization, total DNA from the intact colon, including host colon tissue and the colonic lumen content containing gut microbiota, was purified, then analysed by qPCR using primers for total 16S rRNA gene, *E. coli*-specific 16S rRNA gene, *E. coli decR* (*ybaO*) gene and *A. muciniphila*. The qPCR results were normalized using the mouse *Gapdh* gene*.* The relative abundance of the above genes was also analysed from faeces using total 16S rRNA gene as the loading control. All primer sequences can be found in Supplementary Table 10.

### Methionine diet feeding and allograft experiment in germ-free mice

Six- to eight-week-old germ-free Balb/c mice (Taconic, Balb/cAnNTac (GF), males), with four to eight mice per group, were fed with irradiated CTRL or MR diets, or autoclaved NIH-31 chow diet containing 0.4% methionine or a 1.3% methionine-supplemented chow diet for 3 weeks. They were then inoculated subcutaneously with 2 × 10^5^ mouse colon cancer CT26.CL25 cells and fed with the respective methionine diets for an additional 4–5 weeks.

### Image and immunohistochemistry quantification

The total number of visible tumours in the small intestine of *Apc*^*min+/−*^ mice under different treatments was counted manually. The tumour surface area in the colon and the colonic tissue surface area were quantified in Fiji (ImageJ v.2.1.0/1.53 g) and the final tumour burden was calculated using the total surface area of tumours against the total surface area of the colon.

The mucin-positive areas in the colons of *Apc*^*min+/−*^ mice under control or MR diets were quantified in Fiji using ‘Colour Deconvolution’ with vector of H DAB. The final percentage of positive area was calculated using the total staining area against the total tissue area.

### The UK Biobank data analysis

#### Data

We used a subset (Supplementary Table 1) of the UKB (https://www.ukbiobank.ac.uk/). The UKB participants were recruited starting from 2006. Diet by 24-h recall information (diet24) was collected during on-line cycles 3 (October 2011 to December 2011) and 4 (April 2012 to June 2012), including the estimation of protein intake. We selected these cycles because they include the largest number of individuals who have diet24 information in both cycles. Only existing human data from the UKB were used in this study. The reference ID for Duke IRB Protocol of this study is Pro00109279.

#### Approach

We evaluated the difference in cancer risk between individuals with low-to-normal protein intake versus high protein intake during 24 h (diet24). We considered all cancers combined, except skin cancer and glioblastoma. The ‘risk’ of cancer was defined as the ratio of the number of individuals who have a cancer onset to all individuals in the group.

The low-to-normal protein intake (lowP) was defined as less than 1.0 g of protein consumed per kg of body weight per 24 h. The high protein intake (highP) was defined as more than 1.6 g of protein consumed per kg of body weight per 24 h. Note that definitions of ‘low protein’ and ‘high protein’ intakes vary across studies^44–46^. The age of participants at time of diet24 data collection was between 40 and 75 years, with a modal class (of most frequently seen ages) of between 55 and 70 years in both groups, that is, with ‘low protein’ and with ‘high protein’ intake.

All individuals were divided in two groups: lowP = (<1.0 gram per kilogram of body weight) and highP = (>1.6 gram per kilogram of body weight). Within each group, individuals were further divided into two subgroups: those with cancer onset after 1 July 2012 (after collection of diet24 data was completed), and those without cancer onset after 1 July 2012; that is, all participants (female/male) were categorized into the four groups (Supplementary Table 1).

#### Statistical methods and tools

Fisher’s exact test, confidence intervals for binomial probabilities (Wilson score interval), confidence intervals for the difference of two binomial proportions (Wald interval) and confidence intervals (Wald) for the risk ratio (RR) were used. R standard software packages along with Hmisc and epitools R packages were used.

### Immunoblotting and quantitative real-time PCR

Immunoblotting was performed using anti-Bax (Cell Signaling, cs2772; 1:1,000 dilution), anti-beta-actin (Millipore Sigma, MAB1501; 1:10,000 dilution) or anti-GAPDH (Millipore Sigma CB1001; 1:2,000 dilution) and scanned on an Odyssey imaging system.

Total RNA was purified from tissues using an RNeasy Mini kit (Qiagen) followed by cDNA synthesis with the High-Capacity cDNA Reverse Transcription Kit (Thermo Fisher Scientific). Real-time PCR was performed using iQ SYBR Green Supermix (Bio-Rad). Specific primers are listed in Supplementary Table 10.

### Flow cytometry analysis

Blood samples were collected with EDTA-coated tubes. Red blood cells were lysed with ACK lysis buffer at room temperature for 10 min. The collected lymphocytes were incubated with anti-mouse CD16/CD32 at room temperature for 10 min to block the IgG Fc receptors. Expression of surface markers was detected by simultaneously staining with the following antibodies (eFluor 450 anti-mouse CD45, APC-Cy7 anti-mouse CD4, FITC anti-mouse CD4, FITC anti-mouse CD3, PE-Cy7 anti-mouse CD3, FITC anti-mouse CD8, PerCP-Cy5.5 anti-mouse CD8, PerCP-Cy5.5 PE anti-mouse CD279 (PD-1) and BV711 anti-mouse Tim3) and LIVE/DEAD fixable Aqua dead cell stain kit (Thermo Fisher, L34957) at room temperature for 15 min followed by flow cytometry.

For the IFN-γ and tumour necrosis factor staining, after performing the cell surface staining, the cells were first fixed using BD Cytofix solution (554655) then permeabilized using BD Perm/Wash buffer (554723) and stained with the corresponding antibodies. Flow cytometric analysis was performed on BD LSRFortessa instrument (BD Biosciences) and analysed using FACSDiva (BD Biosciences) or FlowJo V10 (FlowJo) software. All antibodies are listed in Supplementary Table 11. The FACS gating strategy is shown in Supplementary Fig. 6.

### qPCR analysis of faecal microbial sulfur metabolic enzymes

To validate and further analyse the impact of dietary methionine on the expression of faecal microbial sulfur metabolic enzymes, we designed qPCR primers based on the consensus sequences of all known transcripts of each microbial sulfur metabolic gene. Five primer sets for *cysJ*, *cysK*, *cysM*, *dmsA* and *cth* were validated and used in the subsequent qPCR analysis. The qPCR results were normalized against total bacterial 16S rRNA gene. All primer sequences can be found in Supplementary Table 10.

### Lead sulfide assay for faecal hydrogen sulfide production

H_2_S production of mouse faecal samples was detected by the lead sulfide method described by Hines and Mitchell^47^. Faecal samples were collected into 1.5-ml tubes and weighed. PBS was added to each tube and samples were homogenized by vortexing and pipetting. An equal amount of faecal content from each sample was then suspended in PBS supplemented with 400 μM or 10 mM cysteine and 1 mM pyridoxal-5-phosphate. Then, 150 μl of each sample was plated in 96-well plates. A piece of 703-style Whatman filter paper (VWR), soaked in 20 mM lead acetate (Sigma) and dried, was placed over the plate wells and covered with the plate lid with a heavy object on the top. The plate was incubated at 37 °C for 18 h. The formation of lead sulfide indicated by the dark circles on the filter paper was recorded with a digital camera. The relative intensities of lead sulfide were quantified using Fiji 2.0.

### In vitro lymphocyte activation analysis by faecal bacteria

To test whether faecal microorganisms from C57BL/6J mice fed with different methionine diets have distinct abilities to activate T cells, whole blood collected from naive C57BL/6J mice fed with chow diet was treated with 1× RBC Lysis Buffer (Invitrogen, 00-4300-54) for 15 min to lyse red blood cells. The resulting PBMCs were cultured in RPMI 1640 + 10% FBS in a 12-well plate at 1 × 10^6^ cells per well overnight. The next day, five fresh faecal pellets were collected from C57BL/6J mice fed with either control diet or MR diet, washed with 1× PBS three times. The washed faecal microorganisms were then added to the cultured PBMCs with a 1:1,000 ratio of bacteria:cells and incubated for an additional 6 or 12 h. The same amount of PBMCs incubated with CD3/CD28 beads and PMA/ionomycin (BioLegend, 423301) were used as a positive control. PBMCs without any treatment were used as a negative control. The fraction of IFN-γ^+^CD8^+^ T cells was analysed by flow cytometry.

### Seahorse analysis

To analyse the impact of methionine and H_2_S donor on glycolysis, the extracellular acidification rate was measured in a Seahorse XFe96 Analyzer (Seahorse Biosciences) in immune cells treated with different conditions. Specifically, human PBMCs (from STEMCELL) cultured in RPMI 1640 + 10% FBS and 1% penicillin–streptomycin were activated with 300 IU ml^−1^ recombinant human IL-2 (PeproTech, 200-02) and anti-CD3/CD28 beads (Gibco, 11141D) at a 3:1 ratio (beads:T cells) for 14 d. Two days after activation, PBMCs were cultured in control RPMI 1640 medium containing 100 μM methionine (CTRL) or an MR medium containing 10 μM methionine with or without 100 μM NaSH overnight. Treated PBMCs were then plated at 2 × 10^5^ per well (*n* = 8–12) onto coated XF96 cell plates in 40 μl XF RPMI assay medium at pH 7.4. After adding an additional 140 μl XF assay medium, cells were incubated for 1 h at 37 °C without CO_2_. The XF measurements included serial injections of glucose (10 mM), oligomycin (1 μM) and 2-DG (50 mM). The XF assay protocol consisted of 12 measurement cycles, and each cycle was 6 min, including 3 min of mixing, 0 min of waiting and 3 min of measuring. After the Seahorse assay, the remaining assay media from each well were removed without disturbing the cells and protein concentration was measured by BCA (Pierce, 23227) assay. The final glycolysis, glycolytic capacity and glycolytic reserve were normalized to total cell protein contents.

### GAPDH activity assay and sulfhydration analysis

The activity of GAPDH in activated human PBMCs cultured in different media was measured in total cell lysates with the GAPDH activity assay kit based on manufacturer’s instruction (Millipore Sigma, MAK277-1KT).

The sulfhydration of GAPDH was detected using the modified biotin switch assay described by Paul and Snyder^48^. Briefly, activated human PBMCs cultured in different media were homogenized in HEN buffer (250 mM HEPES–NaOH, pH 7.7, EDTA 1 mM and neocuproine 0.1 mM) with 0.1% SDS and 0.1% sodium deoxycholate containing 1× Protease inhibitors and phosphatase inhibitors single-use cocktail (Thermo Fisher, 78443). Next, 100 µg of cell lysates was added to the blocking buffer (HEN buffer with 2.5% SDS, and 20 mM methyl methanethiosulfonate (MMTS)) and incubated at 50 °C for 20 min with rotation on a thermomixer at 1,400 r.p.m. The MMTS was removed by acetone precipitation at −20 °C for 20 min. The protein pellets were resuspended in HENS buffer (HEN buffer with 1% SDS; Thermo Fisher, 90106) and incubated with 0.8 mM Biotin-HPDP (Thermo Fisher, 21341) on a rotator in the dark at room temperature for 75 min. The biotinylated proteins were acetone precipitated, resuspended in HENS buffer and incubated with streptavidin agarose (Thermo Fisher, 20347) with rotation at 4 °C overnight. The streptavidin-bound complexes were washed six times in HENS buffer, and eluted with 2× Laemmli sample buffer (Bio-Rad, 1610747) by boiling at 95 °C for 5 min. The eluted protein was subjected to western blot analysis with anti-GAPDH (Millipore Sigma, CB1001; 1:2,000 dilution).

### Statistics and reproducibility

Values are expressed as the mean ± s.e.m. from at least three biological replicates, unless otherwise indicated in the figure legend. Differences between the means with two comparison groups were analysed by two-tailed, unpaired Student’s *t*-test^49^ and *P* values were reported. Differences between the means with more than two comparison groups were analysed by either Kruskal–Wallis test or two-way ANOVA with correction for multiple comparisons by controlling the false discovery rate and adjusted *P* values (*q* values) were reported. Data were analysed using Prism Software 8.0 (GraphPad). For all in vivo experiments, outlier samples that were below Q1 minus 3.0 times the IQR or above Q3 plus 3.0 times the IQR were removed.

For animal studies, the sample size in each independent experiment was estimated to achieve 30% of the tumour size/weight difference with 80% of power (syngeneic tumour models) or to achieve a twofold difference of tumour number/burden with 80% of power (*Apc*^*min+/−*^ model or AOM/DSS CRC model). For gene expression analysis, the sample size was estimated to achieve a twofold difference of gene expression level with 80% of power. In vitro cell culture experiments were independently performed at least three times and similar results were observed. Each independent experiment was performed with at least three biological replicates, and no explicit calculations were done to determine the sample size.

Additional methods on RNA-seq, scRNA-seq, bacterial 16S rRNA gene amplicon sequencing, faecal bacterial RNA-seq (metatranscriptomics) and faecal metabolites by liquid chromatography–mass spectrometry (LC–MS) are available in the Supplementary Information.

### Reporting summary

Further information on research design is available in the Nature Portfolio Reporting Summary linked to this article.

### Supplementary information

Supplementary Information Supplementary Methods and Figs. 1–6.

Reporting Summary

Supplementary Tables 1–11

Source Data for Supplementary Fig. 3 Tumour weight of subcutaneous CT26.CL25 tumours in germ-free Balb/c mice repopulated with different bacteria.

### Source data

Source Data Fig. 1 Numerical source data.

Source Data Fig. 2 Numerical source data.

Source Data Fig. 3 Numerical source data.

Source Data Fig. 4 Numerical source data.

Source Data Fig. 5 Numerical source data.

Source Data Fig. 6 Numerical source data.

Source Data Fig. 6 Unprocessed immunoblots for Fig. 6f.

Source Data Fig. 7 Numerical source data.

Source Data Fig. 8 Numerical source data.

Source Data Extended Data Fig. 1 Numerical source data.

Source Data Extended Data Fig. 1 Unprocessed immunoblots for Extended Data Fig. 1b.

Source Data Extended Data Fig. 2 Numerical source data.

Source Data Extended Data Fig. 3 Numerical source data.

Source Data Extended Data Fig. 4 Numerical source data.

Source Data Extended Data Fig. 5 Numerical source data.

Source Data Extended Data Fig. 6 Numerical source data.

Source Data Extended Data Fig. 7 Numerical source data.

Source Data Extended Data Fig. 8 Numerical source data.

Source Data Extended Data Fig. 9 Numerical source data.

## Data Availability

The RNA-seq dataset has been deposited to the Gene Expression Omnibus under accession number GSE165993. The scRNA-seq dataset has been deposited to the Gene Expression Omnibus under accession number GSE181220. The metatranscriptomic data have been submitted to the Sequence Read Archive under accession number PRJNA892072. Additional information about differentially expressed genes of RNA-seq data and IPA results is included in Supplementary Tables 2 and 3. The 16S rRNA amplicon sequencing results are in Supplementary Table 4. The scRNA-seq results on immune cell clusters and T cell populations are in Supplementary Tables 5–7. The faecal metabolite data are available in Supplementary Table 8. The list of active transcripts of H2S-producing enzymes is in Supplementary Table  9. All primers and antibodies used in the study are in Supplementary Tables 10 and 11. Databases used for faecal metabolite annotation were as follows: mzCloud Advanced Mass Spectral Database (mzCloud, Thermo Fisher Scientific; https://www.mzcloud.org/); NIST 2020 LC–MS/MS library (NIST library, National Institute of Standards and Technology; https://www.nist.gov/programs-projects/nist20-updates-nist-tandem-and-electron-ionization-spectral-libraries); and ChemSpider chemical structure database (ChemSpider; http://www.chemspider.com/). Source data are provided with this paper.
