# Supplementary material for: Methionine restriction-induced sulfur deficiency impairs antitumour immunity partially through gut microbiota
Source: Nat Metab. 2023 Aug 3;5(9):1526–43. doi: 10.1038/s42255-023-00854-3 (PMC10513933; doi:10.1038/s42255-023-00854-3)
Supplement: Supplementary file 1 — Supplementary Methods and Figs. 1–6. [file 42255_2023_854_MOESM1_ESM.pdf]

nature metabolism

Article https://doi.org/10.1038/s42255-023-00854-3

Methionine restriction-induced sulfur deficiency impairs antitumour immunity partially through gut microbiota

In the format provided by the authors and unedited

Supplementary Methods

RNA-seq analysis

RNA was extracted from subcutaneously grafted CT26.WT tumors of mice with QIAzol, and purification of total RNA was performed with the QIAGEN RNeasy RNA isolation kit (Redwood City, CA) according to the manufacturer's protocol. RNA-seq libraries were generated with 1 μg of RNA as input using the TruSeq RNA Sample Prep Kit (Illumina, San Diego, CA) with poly(A)-enrichment according to the TruSeq protocol. Index multiplexed samples were sequenced using the 75-bp single-end protocol via the NextSeq500 (Illumina) according to the manufacturer's protocol. Reads (30–60 million reads per sample) were aligned to the University of California Santa Cruz (UCSC) mm10 reference genome using STAR (Version 2.6) with Gencode vM23 annotation. The quantification results from "featureCount (subread, Version 1.4.6)" were then analyzed with the Bioconductor package DESeq2, which fits a negative binomial distribution to estimate technical and biological variability. Comparisons were made between experimental conditions and a gene was considered differentially expressed when the p value for differential expression was less than 0.01.

Significant gene lists were subjected to Ingenuity Pathway Analysis (IPA, QIAGEN, USA; Content version 60467501). GSEA was performed using GSEA software (Version 4.1). Genes were pre-ranked based on the fold change of gene expression. This application scores a sorted list of genes with respect to their enrichment of selected functional categories (Kyoto Encyclopedia of Genes and Genomes [KEGG], Biocarta, Reactome, and gene ontology [GO]). The significance of the enrichment score was assessed using 1000 permutations. Benjamini and Hochberg's false discovery rate (FDR) was calculated for multiple testing adjustments. q-value < 0.05 was considered significant. The resulting enriched pathways were visualized using the Cytoscape (Version 3.8.2) Enrichment Map plugin.

scRNA-seq analysis of small intestinal CD45^{+} immune cells

Isolation of CD45^{+} immune cells from the small intestine

To analyze total CD45^{+} immune cells from diet-fed C57BL6/J donor mice and fecal transplanted *Apc*$^{min+/-}$ mice, mice were euthanized and surface-sterilized using 70% (vol/vol) ethanol. After removing the mesentery/fat and Peyer`s patches, the small intestine was flashed with ice cold PBS ($Mg^{2+}Ca^{2+}$ free), then opened longitudinally and removed the mucous layer by gently rubbing the intestine between fingers in cold PBS. The small intestine was cut into 0.5-1cm pieces and washed twice in 20 ml PBS ($Mg^{2+}Ca^{2+}$ free) containing 0.37 mg/ml EDTA (1.25 mM) and 0.145 mg/ml DTT (1 mM) in a shaking incubator at 37°C for 15 min, and then twice with 5 ml ice cold PBS ($Mg^{2+}Ca^{2+}$ free) by gently shaking. The remaining tissue was then digested by incubating in digestion medium (20 ml RPMI-1640 containing 5% fetal calf serum, 20 mM HEPES, 100 U/ml penicillin /streptomycin, 1 mg/ml Collagenase I (Sigma-Aldrich, C0130), 1 mg/ml Dispase II (Sigma, D4693) and 20 µg/ml DNase I (Roche, 11284932001)) for 15 - 20 min at 37°C while gently shaking, the liberated cells in the supernatant were collected and placed on ice. Repeat the digestion step for 2 additional times with 10-15 ml fresh digestion medium each time. Following the third digestion step all fractions were combined and passed through a 70 μm strainer (Corning, 431751). The filtered single cells were collected,

washed with 10 mL ice cold sterile PBS (without FCS) once, and resuspend in 100 μl ice cold sterile PBS. The resulting single cell suspension from two mice in each group was combined and stained for $CD45^+$ and PI, and sorted by flow cytometry. The final $CD45^+$ single cells were used for generating scRNA-seq libraries.

scRNA-seq library preparation and sequencing

The cells were counted and examined for viability with trypan blue staining using a TC-20 cell counter (Bio-Rad). Approximately 10,000 live cells with above 70% viability were loaded into the Single Cell Chip to generate single cell emulsion in Chromium Controller (10x Genomics, 120263) with Chromium Single Cell 3' Library & Gel Bead Kit v3.1 (10x Genomics, 1000268). Reverse transcription of mRNA and cDNA amplification were carried out following the manufacture's instruction (10x Genomics, 1000268). The amplified cDNA was further fragmented to construct NGS libraries. The libraries were then sequenced by the NIEHS Epigenomics and DNA Sequencing Core Laboratory with the parameters recommended in the manufacture's instruction.

scRNA-seq data processing

Raw read processing was carried out using the Cell Ranger Single-Cell Software Suite (version 6.0.1, 10x Genomics Inc., CA). Briefly, the demultiplexed FASTQ files (paired-end, Read 1: 30bp, Read 2: 100bp) were generated using the CellRanger *mkfastq* command. The primary data analyses which included alignment, filtering, barcode counting and UMI quantification for determining gene transcript counts per cell (generated a gene-barcode matrix), quality control, clustering and statistical analysis were performed using CellRanger *count* command with genome reference "refdata-gex-mm10-2020-A".

Raw gene expression matrices generated per sample by CellRanger were imported into R (version 4.0.1) and converted to a Seurat object using the Seurat R package (version 4.0.2) [1]. Function *decontX* from R package "celda" [2] was used to remove RNA contamination. Dead cells and doublets were also removed. Low quality cells were further filtered out based on the following steps. First, the total number of UMIs and genes, and percentage of UMIs derived from mitochondrial genome for each cell were counted. Then, the upper bound was calculated as mean plus two standard deviation (SD) and the lower bound as mean minus two SD for both the total UMIs and genes, respectively. Next, cells which had over 10% UMIs derived from mitochondrial genome were discarded. Finally, cells with total UMIs or genes outside of the upper and lower bounds were removed.

Single-cell gene expression quantification and major cell type classification

For the remaining cells (cleaned cells), Seurat *SCTransform* function was used for gene expression normalization, highly variably genes (HVG) and scaling. Gene expression data were normalized to total cellular read count(10000/cell); and the top 3000 highly variably genes (HVGs) were used as features for dimensionality reduction and clustering. Cell-Cycle scores were also calculated using Seurat *CellCycleScoring* function from a set of cell-cycle related gene pairs as input to classify cells into G1, S and G2/M phase. Cell cycle effect was regressed out using *SCTransform* function as well. The Seurat *RunPCA* functions was performed to calculate PCA and top 40 principal components (PCs) selected for downstream analysis. After R package "Harmony" [3] corrected the batch effects, the Seurat FindNeighbors function was used to construct a Shared Nearest Neighbor (SNN) Graph, and the RunUMAP function was then applied to plot the selected

significant PCs. FindClusters function with “resolution = 1” parameter was carried out to cluster cells into different groups.

To identify marker genes for each cluster or cell type, the Seurat *FindMarkers* function was applied to compare the gene expression values of cells from the cluster of interest to that of cells from the rest of clusters. Cell clusters were annotated by the following two approaches: 1) the canonical marker genes were applied to annotated cell clusters to known biological cell types; 2) the predicted marker genes were applied for each cluster. The predicted marker genes could further confirm the annotated cell type, at the same time, they could also be used to name cell clusters which don’t have canonical marker genes.

Seurat *FindMarkers* function with parameter of “MAST” [4] test was used to calculate DEG between two group cells of interest. The final DEG lists in Supplementary Table 7 were generated using cut offs of padj<0.05 and/or |FC|>1.3. The downregulated pathways enriched in intestinal CD8 effector cells in response to diet feeding or fecal transplantation were analyzed using the corresponding DEG lists in https://biit.cs.ut.ee/gprofiler/gost.

Analysis of T cell subpopulation

To further explore features of T cells, we carried out a series of analyses focusing on $CD8^+$ and $CD4^+$ T cells. The T cells were extracted from whole single cell population based on the cell barcodes. Then, gene expression matrix of these T cells was obtained from cleaned data. The downstream analyses, including normalization, HVG selection, scaling, PCA calculation, batch effect correction, clustering and DEG calling were applied following the same procedure as single cell analysis described in previous section.

Fecal bacterial 16S rRNA gene amplicon sequencing (UNC Microbiome Core for *Apc*$^{min+/-}$ mice)

Stool Samples DNA Isolation

Samples were transferred to a 2 ml tube containing 200 mg of ≤106 µm glass beads (Sigma, St. Louis, MO) and 0.5 ml of Qiagen PM1 buffer (Valencia, CA). Bead beating was performed for 5 minutes in a Qiagen TissueLyser II at 30Hz. After centrifugation for 5 minutes, 0.45ml of supernatant was transferred to a new tube containing 0.15ml of Qiagen IRS solution followed by incubation at 4°C overnight. After a brief centrifugation, supernatants were transferred to deep well plates containing 0.45ml of Qiagen binding buffer supplemented with Qiagen ClearMag Beads. DNA was purified using the automated KingFisher™ Flex Purification System and eluted in DNase free water [5-7].

16S rRNA gene amplicon sequencing

12.5 ng of total DNA was amplified using universal primers targeting the V4 region of the bacterial 16S rRNA gene. Primer sequences contained overhang adapters appended to the 5’ end of each primer for compatibility with Illumina sequencing platform. The primers used were F515/R806 [8]. Master mixes contained 12.5 ng of total DNA, 0.5 µM of each primer and 2x KAPA HiFi HotStart ReadyMix (KAPA Biosystems, Wilmington, MA). Each 16S rRNA gene amplicon was purified using the AMPure XP reagent (Beckman Coulter, Indianapolis, IN). In the next step each sample was amplified using a limited cycle PCR program, adding Illumina sequencing adapters and dual-index barcodes (index 1(i7) and index 2(i5)) (Illumina, San Diego, CA) to the amplicon target. The final libraries were again purified using the AMPure XP reagent (Beckman Coulter), quantified and normalized prior

to pooling. The DNA library pool was then denatured with NaOH, diluted with hybridization buffer and heat denatured before loading on the MiSeq reagent cartridge (Illumina) and on the MiSeq instrument (Illumina). Automated cluster generation and paired–end sequencing with dual reads were performed according to the manufacturer's instructions.

Bioinformatics Analysis

Sequencing output from the Illumina MiSeq platform were converted to fastq format and demultiplexed using Illumina Bcl2Fastq 2.18.0.12. The resulting paired-end reads were processed using QIIME 2 2018.11 [9]. Index and linker primer sequences were trimmed using the QIIME 2 invocation of cutadapt. The resulting paired-end reads were processed with DADA2 through QIIME 2 including merging paired ends, quality filtering, error correction, and chimera detection[10]. Amplicon sequencing units from DADA2 were assigned taxonomic identifiers with respect to Green Genes release 13_08 using the QIIME 2 q2-featureclassifier[11]. Alpha diversity with respect to: Faith PD whole tree, Evenness (Shannon) index, and observed species number metrics; was estimated using QIIME 2 at a rarefaction depth of 5,000 sequences per subsample. Beta diversity estimates were calculated within QIIME 2 using weighted and unweighted Unifrac distances as well as Bray-Curtis dissimilarity between samples at a subsampling depth of 5,000. Results were summarized, visualized through principal coordinate analysis, and significance was estimated as implemented in QIIME 2. Significance of differential abundance was estimated using ANCOM as implemented in QIIME 2 [12].

Small intestinal and fecal bacterial 16S rRNA gene amplicon sequencing (NIEHS Epigenomic Core for B6 mice)

Bacterial DNA Isolation

Frozen small intestine samples or feces were pulverized in liquid nitrogen using mortar and pestle. 50-100 mg small intestinal samples or 1-2 fecal pellets were lysed by bead beating with 7 mm steel beads for 5 minutes in a Qiagen TissueLyser II at 50Hz in InhibitEX Buffer, centrifuged at 21,130 g. 350 μl supernatant was used for DNA isolation with QIAamp® Fast DNA Stool Mini kit (Qiagen) on a QIAcube instrument according to manufacturer's instructions.

16S rRNA gene amplicon sequencing

Sequencing libraries were prepared as described in above "Fecal bacterial 16S rRNA gene amplicon sequencing" but using primers amplifying V3 region for small intestinal samples [13] or V3/V4 region for fecal samples [14]. Primer sequences are in Supplementary Table 10.

Fecal metabolite analysis (Duke platform)

Sample preparation for LC-MS

20-30 mg of feces collected from C57BL/6J mice fed with 0.86% control diet or 0.172% methionine restricted diet were extracted on ice for 15 minutes with 80% methanol/20% water pre-cooled at -80 °C (72 μl per 1 mg of dry feces). Extracts were then transferred into microcentrifuge tubes and centrifuged at 16,100 g for 10 minutes. The resulting supernatants were transferred to new tubes and dried in a vacuum concentrator at room temperature. The dry pellets were reconstituted into 30 μl sample solvent (water:methanol:acetonitrile, 2:1:1, v/v) and 3 μl was further analyzed by liquid chromatography-mass spectrometry (LC-MS).

LC-MS method

Ultimate 3000 UHPLC (Dionex) is coupled to Q Exactive Plus-Mass spectrometer (QE-MS, Thermo Scientific) for metabolite profiling. A hydrophilic interaction chromatography method (HILIC) employing an Xbridge amide column (100 x 2.1 mm i.d., 3.5 μm; Waters) is used for polar metabolite separation. Detailed LC method was described previously [15] except that mobile phase A was replaced with water containing 5 mM ammonium acetate (pH 6.8). The QE-MS is equipped with a HESI probe with related parameters set as below: heater temperature, 120 °C; sheath gas, 30; auxiliary gas, 10; sweep gas, 3; spray voltage, 3.0 kV for the positive mode and 2.5 kV for the negative mode; capillary temperature, 320 °C; S-lens, 55; A scan range (m/z) of 70 to 900 was used in positive mode from 1.31 to 12.5 minutes. For negative mode, a scan range of 70 to 900 was used from 1.31 to 6.6 minutes and then 100 to 1,000 from 6.61 to 12.5 minutes; resolution: 70000; automated gain control (AGC), 3×10^6 ions. Customized mass calibration was performed before data acquisition. Metabolomics data analysis- LC-MS peak extraction and integration were performed using Sieve 2.2 (Thermo Scientific). The peak area was used to represent the relative abundance of each metabolite in different samples. Missing values were handled as described in [15]. MetaboAnalyst package was used for the analysis of the metabolomics data [16].

Fecal metabolite analysis (UNC platform)

Sample preparation for LC-MS

Fecal pellets collected from C57BL/6J mice fed with 0.86% control diet or 0.172% methionine restricted diet were thawed and weighed. The fecal pellet sample was added with a weight-adjusted volume (100 μL per 30 mg) of water and a stainless steel bead (5 mm diameter) in a microcentrifuge tube for homogenization with a TissueLyzer at 50 Hz for 10 minutes. Weight-adjusted volumes of methanol (400 μL per 30 mg) was then added to each of the sample to precipitate proteins under 4°C for 1 hour, and 400 μL of the supernatant was transferred to a new microcentrifuge tube and dried with SpeedVac® after centrifugation at 15,000 ×g for 10 minutes. The dried samples were reconstituted with 150 μL of 2% acetonitrile in water, centrifuged at 15,000 ×g for 10 minutes, and the supernatant is transferred to a glass vial ready for LC-MS analysis.

LC-MS method

The LC-MS analysis was facilitated with a Thermo Fisher Scientific Vanquish UHPLC coupled to a Q Exactive mass spectrometer with a HESI as the interface, and the method applied was previously reported[17,18]. The fecal analytes were injected (3 μL) into a Waters Acquity UPLC HSS T3 (reverse phase C18, 100 Å, 1.8 μm, 2.1 mm × 100 mm) analytical column controlled at 40 °C, with the mobile phase composed of water (A) and acetonitrile (B) both with 0.1% formic acid at a flow rate of 0.4 mL/min. The 15-min-gradient for chromatographic separation was set as the following: 2% B from 0-1 min; 2%-15% B from 1-3 min; 15%-50% B from 3-6 min; 50%-98% B from 6-7.5 min; 98% B held from 7.5-11.5 min; 98%-2% B from 11.5-11.6 min; and 2% B held from 11.6-15 min for a final re-equilibration. The mass spectrometry was set to scan under the positive mode with the sheath gas, auxiliary gas, and sweep gas set to flow rates of 50, 13, and 3 psi, respectively. With the spray voltage set to 3.5 kV, the capillary and auxiliary gas heating temperature were respectively controlled to 263°C and 425°C to fully scan across *m/z* 70 to 1,000. The resolution was set to 70,000 FWHM (*m/z* 200). The AGC and the maximal

injection time (MIT) was set to $2×10^5$ and 50 msec, respectively. Routine mass calibrations were conducted before and after the sample analysis. The samples were blocked-randomized for the injection order. Quality control samples were prepared by pooling the aliquots of each of the sample, and standard solutions composed of [D_3]-tryptophan, [D_5]-glutamic acid, and [D_2]-indole propionic acid, each with the concentration as 500 nM, was prepared to monitor the performance of the LC-MS across the sample analysis, which the results showed satisfying LC-MS performance. Quality control samples, standard solutions, and blanks (using 0.1% formic acid in water) were regularly injected in parallel to the samples to assure the instrumental quality. If MS/MS spectrums were to be collected, the parallel reaction monitoring (PRM) mode was used, with the isolation width, AGC, and MIT set to 1.2, $3×10^5$ and 100 msec, respectively, at the resolution of 17,500 FWHM (*m/z* 200).

Data processing

Data obtained from above LC-MS analysis were processed and analyzed as described recently [19]. Specifically, LC-MS data were converted and centroided to mzXML files with ProteoWizard (ver 3), then processed through two stages: feature identification and compound characterization.

Feature annotation was performed using XCMS (ver 3.16.1) in assistance of the IPO package (ver 1.20.0), as referenced in past studies[20-24]. First, the parameters in the algorithms of XCMS, including feature detection (*centWave)*, retention time adjustment (*obiwarp*), and correspondence (*density*), were optimized with the data from the quality control samples using the IPO package. Next, all LC-MS data were processed with XCMS optimized parameters to obtain a list of molecular features with distinct *m/z*-RT signatures. For subsequent analysis, features detected in 50% of the CTRL or the MR group were used and missing values in the measurement matrix were imputated with random forest algorithm by the missForest package (ver.1.4) as suggested in previous comparison studies [25-27]. These features were then log-transformed, analyzed by Feature-wise Student's *t* test, and prioritized by their *p*-values adjusted for false discovery (*q*-values) and the fold-change between CTRL and MR groups. A prioritized list of 1,100 features were used to build inclusion lists to capture their MS/MS spectrums to maximize the efficiency of compound annotation.

Compound characterization was performed in Compound Discoverer using a previously described workflow [28]. First, the chemical formulas were matched after clustering the features into unknown compounds. Then, the matched formula was queried against four annotation databases: our in-house LC-MS/MS library, mzCloud Advanced Mass Spectral Database (mzCloud, Thermo Fisher Scientific), NIST 2020 LC-MS/MS library (NIST library, National Institute of Standards and Technology, MD), and ChemSpider chemical structure database (ChemSpider). Our in-house library was built by testing authenticated standards of 734 common metabolites. mzCloud and NIST library are public or commercially available spectrum databases constructed with experimentally fragmented ions. ChemSpider unites multiple data sources to provide curated structural data for Compound Discoverer to simulate *in silico* fragmentation. Any match, from the different annotation sources, was considered as a reliable compound characterization only when it fulfilled at least two of the three orthogonal criteria: the retention time (± 0.25 min), accurate mass (± 5 ppm), and fragment ion pattern (each

fragment with ± 10 ppm). The final characterized compounds include those presented in Fig. 4a and Supplementary Table 8c.

Fecal bacterial RNA-seq and metatranscriptomic analysis

Fecal RNA was isolated with Rneasy PowerMicrobiome Kit (Qiagen) and cDNA libraries prepared with TruSeq RNA Stranded with Ribo Zero plus kit (Illumina). Index multiplexed samples were sequenced using the 75-bp single-end protocol on the Novaseq 600 instrument (Illumina) according to the manufacturer protocol.

Sequencing reads were trimmed using fastp [29] (v0.20.1) with the default settings. The reads were then decontaminated with bbduk (https://sourceforge.net/projects/bbmap/, v38.84, hdist=1 k=60 -Xmx500g) by filtering out reads that mapped to the *Mus musculus* GRCm39 reference genome transcripts (GCF_000001635.27, accessed May 4, 2022). To determine the activity of sulfur metabolic genes, 500 protein sequences were sampled from each of 87 sulfur metabolic gene hidden Markov models (HMMs) described previously [30] and reverse translated to nucleotide sequences, resulting in 43500 sampled sulfur genes. These sampled genes were indexed using kallisto index [31] (v.0.46.2 -k 15) for gene count estimation. Then, the trimmed, decontaminated reads were pseudomapped to the sampled sulfur genes index using the default settings for kallisto quant. For each sulfur gene, the gene counts were estimated to be the sum of pseudomapped reads across the 500 sampled sequences. In addition, the counts were combined for HMMs that model the same metabolic gene. Differential expression analysis was performed using R DESeq2 package [32] (v1.34.0), comparing the methionine-restricted diet to the control diet. P values were adjusted using Benjamini-Hochberg method to calculate the false discovery rate. Genes with an adjusted p-value less than 0.05 were taken to be significantly differentially expressed. To accurately compare gene count estimations, the FPKM for the significantly differentially expressed genes was calculated using the fpkm DESeq2 function.

References:

1 Butler, A., Hoffman, P., Smibert, P., Papalexi, E. & Satija, R. Integrating single-cell transcriptomic data across different conditions, technologies, and species. *Nat Biotechnol* **36**, 411-420, doi:10.1038/nbt.4096 (2018).

2 Campbell, J. *et al.* celda: CEllular Latent Dirichlet Allocation. *R package version 1.8.1.* (2021).

3 Korsunsky, I. *et al.* Fast, sensitive and accurate integration of single-cell data with Harmony. *Nat Methods* **16**, 1289-1296, doi:10.1038/s41592-019-0619-0 (2019).

4 Finak, G. *et al.* MAST: a flexible statistical framework for assessing transcriptional changes and characterizing heterogeneity in single-cell RNA sequencing data. *Genome Biol* **16**, 278, doi:10.1186/s13059-015-0844-5 (2015).

5 Imane Allali, J. W. A., Jeffrey Roach, Maria Belen Cadenas, Natasha Butz, Hosni M. Hassan, Matthew Koci, Anne Ballou, Mary Mendoza, Rizwana Ali & M. Andrea Azcarate-Peril A Comparison of Sequencing Platforms and Bioinformatics Pipelines for Compositional Analysis of the Gut Microbiome. *BMC Microbiology* **17** (2017).

6 M. Andrea Azcarate-Peril, N. B., Maria Belen Cadenas, Matthew Koci, Anne Ballou, Mary Mendoza, Rizwana Ali, Hosni Hassan & Schottel, J. L. An Attenuated Salmonella enterica Serovar Typhimurium Strain and Galacto-Oligosaccharides Accelerate Clearance of Salmonella Infections in Poultry through Modifications to the Gut Microbiome. *Applied Environmental Microbiology* **84**, e02526-02517, doi:10.1128/AEM.02526-17 (2018).

7 Lucía Guadamuro, M. A. A.-P., Rafael Tojo, Baltasar Mayo, and Susana Delgado. Use of high throughput amplicon sequencing and ethidium monoazide dye to track microbiota changes in an equol-producing menopausal woman receiving a long-term isoflavones treatment. *AIMS Microbiol.* **5**, 102–116, doi:10.3934/microbiol.2019.1.102 (2019).

8 J. Gregory Caporaso, C. L. L., William A. Walters, Donna Berg-Lyons, Catherine A. Lozupone, Peter J. Turnbaugh, Noah Fierer, and Rob Knight. Global patterns of 16S rRNA diversity at a depth of millions of sequences per sample. *Proceedings of the National Academy of Sciences of the United States of America* **108**, doi:https://doi.org/10.1073/pnas.1000080107 (2011).

9 Bolyen, E. *et al.* Reproducible, interactive, scalable and extensible microbiome data science using QIIME 2. *Nat Biotechnol* **37**, 852-857, doi:10.1038/s41587-019-0209-9 (2019).

10 Benjamin J Callahan, P. J. M., Michael J Rosen, Andrew W Han, Amy Jo A Johnson & Susan P Holmes. . DADA2: High-resolution sample inference from Illumina amplicon data. *Nature Methods* **13**, 581-583 (2016).

11 Nicholas A. Bokulich, B. D. K., Jai Ram Rideout, Matthew Dillon, Evan Bolyen, Rob Knight, Gavin A. Huttley, and J. Gregory Caporaso. Optimizing taxonomic classification of marker-gene amplicon sequences with qiime 2's q2-feature-classifier plugin. *Microbiome* **6**, 90 (2018).

12 Lin, H. & Peddada, S. D. Analysis of compositions of microbiomes with bias correction. *Nat Commun* **11**, 3514, doi:10.1038/s41467-020-17041-7 (2020).

13 Qin, Y. *et al.* An obesity-associated gut microbiome reprograms the intestinal epigenome and leads to altered colonic gene expression. *Genome Biol* **19**, 7, doi:10.1186/s13059-018-1389-1 (2018).
14 Klindworth, A. *et al.* Evaluation of general 16S ribosomal RNA gene PCR primers for classical and next-generation sequencing-based diversity studies. *Nucleic Acids Res* **41**, e1, doi:10.1093/nar/gks808 (2013).
15 Liu, X., Ser, Z. & Locasale, J. W. Development and quantitative evaluation of a high-resolution metabolomics technology. *Anal Chem* **86**, 2175-2184, doi:10.1021/ac403845u (2014).
16 Chong, J. *et al.* MetaboAnalyst 4.0: towards more transparent and integrative metabolomics analysis. *Nucleic Acids Res* **46**, W486-W494, doi:10.1093/nar/gky310 (2018).
17 Lai, Y. *et al.* High-coverage metabolomics uncovers microbiota-driven biochemical landscape of interorgan transport and gut-brain communication in mice. *Nature Communications* **12**, 6000, doi:10.1038/s41467-021-26209-8 (2021).
18 Hsiao, Y.-C. *et al.* Development of LC-HRMS untargeted analysis methods for nasal epithelial lining fluid exposomics. *Journal of Exposure Science & Environmental Epidemiology*, doi:10.1038/s41370-022-00448-3 (2022).
19 Hsiao, Y. C. *et al.* Untargeted Metabolomics to Characterize the Urinary Chemical Landscape of E-Cigarette Users. *Chem Res Toxicol* **36**, 630-642, doi:10.1021/acs.chemrestox.2c00346 (2023).
20 Cheema, A. K. *et al.* Alterations in Tissue Metabolite Profiles with Amifostine-Prophylaxed Mice Exposed to Gamma Radiation. *Metabolites* **10**, 211 (2020).
21 Fernández-Ochoa, Á. *et al.* A Case Report of Switching from Specific Vendor-Based to R-Based Pipelines for Untargeted LC-MS Metabolomics. *Metabolites* **10**, 28 (2020).
22 Libiseller, G. *et al.* IPO: a tool for automated optimization of XCMS parameters. *BMC Bioinformatics* **16**, 118, doi:10.1186/s12859-015-0562-8 (2015).
23 Tautenhahn, R., Böttcher, C. & Neumann, S. Highly sensitive feature detection for high resolution LC/MS. *BMC Bioinformatics* **9**, 504, doi:10.1186/1471-2105-9-504 (2008).
24 Smith, C. A., Want, E. J., O'Maille, G., Abagyan, R. & Siuzdak, G. XCMS: Processing Mass Spectrometry Data for Metabolite Profiling Using Nonlinear Peak Alignment, Matching, and Identification. *Analytical Chemistry* **78**, 779-787, doi:10.1021/ac051437y (2006).
25 Kokla, M., Virtanen, J., Kolehmainen, M., Paananen, J. & Hanhineva, K. Random forest-based imputation outperforms other methods for imputing LC-MS metabolomics data: a comparative study. *BMC Bioinformatics* **20**, 492, doi:10.1186/s12859-019-3110-0 (2019).
26 Stekhoven, D. J. & Bühlmann, P. MissForest—non-parametric missing value imputation for mixed-type data. *Bioinformatics* **28**, 112-118, doi:10.1093/bioinformatics/btr597 (2011).
27 Wei, R. *et al.* Missing Value Imputation Approach for Mass Spectrometry-based Metabolomics Data. *Scientific Reports* **8**, 663, doi:10.1038/s41598-017-19120-0 (2018).

28 Hao, L. *et al.* Comparative Evaluation of MS-based Metabolomics Software and Its Application to Preclinical Alzheimer's Disease. *Scientific Reports* **8**, 9291, doi:10.1038/s41598-018-27031-x (2018).
29 Chen, S., Zhou, Y., Chen, Y. & Gu, J. fastp: an ultra-fast all-in-one FASTQ preprocessor. *Bioinformatics* **34**, i884-i890, doi:10.1093/bioinformatics/bty560 (2018).
30 Wolf, P. G. *et al.* Diversity and distribution of sulfur metabolic genes in the human gut microbiome and their association with colorectal cancer. *Microbiome* **10**, 64, doi:10.1186/s40168-022-01242-x (2022).
31 Bray, N. L., Pimentel, H., Melsted, P. & Pachter, L. Near-optimal probabilistic RNA-seq quantification. *Nat Biotechnol* **34**, 525-527, doi:10.1038/nbt.3519 (2016).
32 Love, M. I., Huber, W. & Anders, S. Moderated estimation of fold change and dispersion for RNA-seq data with DESeq2. *Genome Biol* **15**, 550, doi:10.1186/s13059-014-0550-8 (2014).

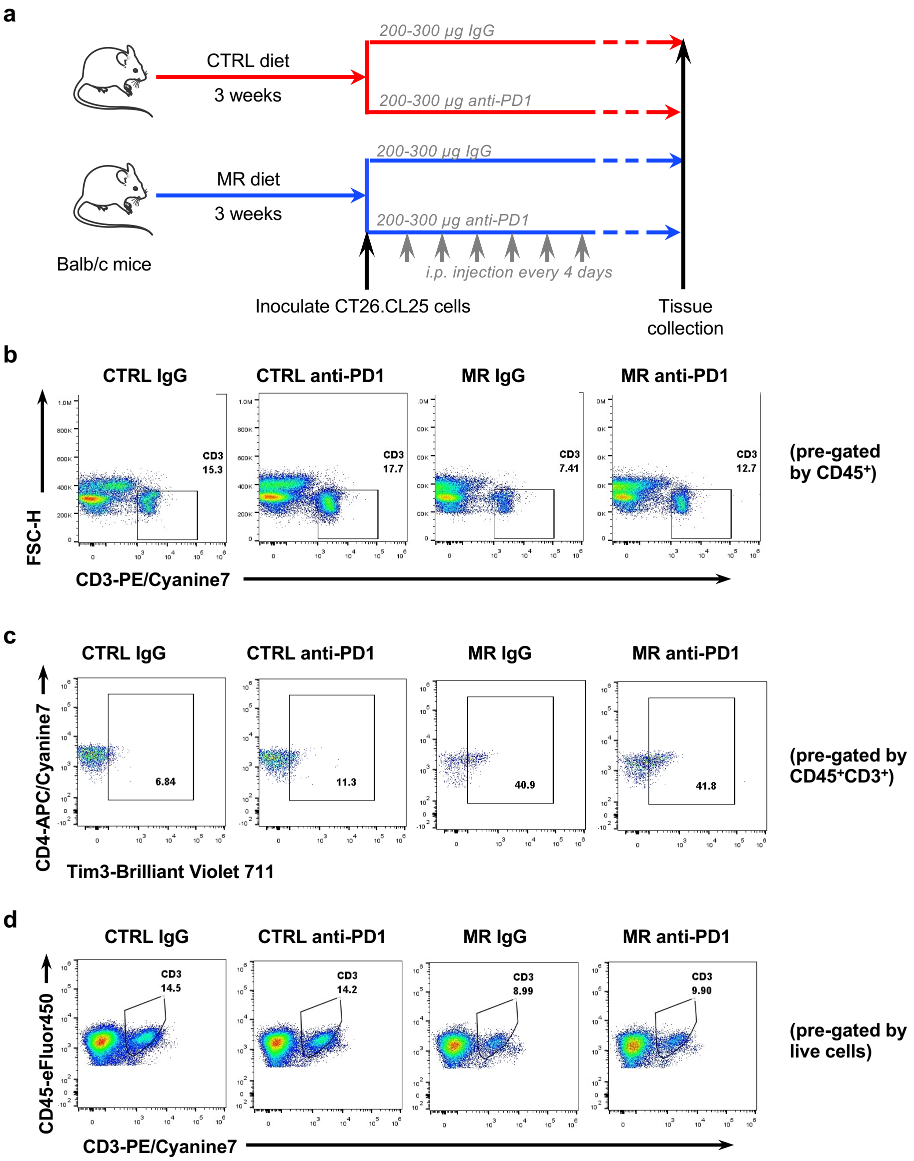

Supplementary Fig. 1. Dietary methionine restriction dampens tumor response to anti-tumor immunotherapy in Balb/c mice.

a, Schematic of the anti-PD1 immunotherapy experiment. Immunocompetent Balb/c mice were fed with either CTRL diet or a MR diet for 3 weeks. After s.c. injected with 2x105 CT26.CL25 cells, mice in each group were then randomly divided with 2 groups to be i.p. injected with 200 or 300 μg control IgG or anti-PD1 antibody every 4 days. Allografted tumors were monitored and analyzed 2-3 weeks after inoculation. **b**, Dietary methionine restriction reduced circulating CD3+ T cells. Representative flow cytometry plots of $CD3^+$ T cells in the blood from indicated treatment groups. **c**, Dietary methionine restriction increases circulating $Tim3^+$ T cells Balb/c mice. Representative flow cytometry plots of $Tim3^+$ $CD4^+$ T cells in the blood from indicated treatment groups. **d**, Dietary methionine restriction also reduced intratumoral $CD3^+$ T cells after the anti-PD1 antibody treatment. Representative flow cytometry plots of $CD3^+$ T cells in the tumors from indicated treatment groups

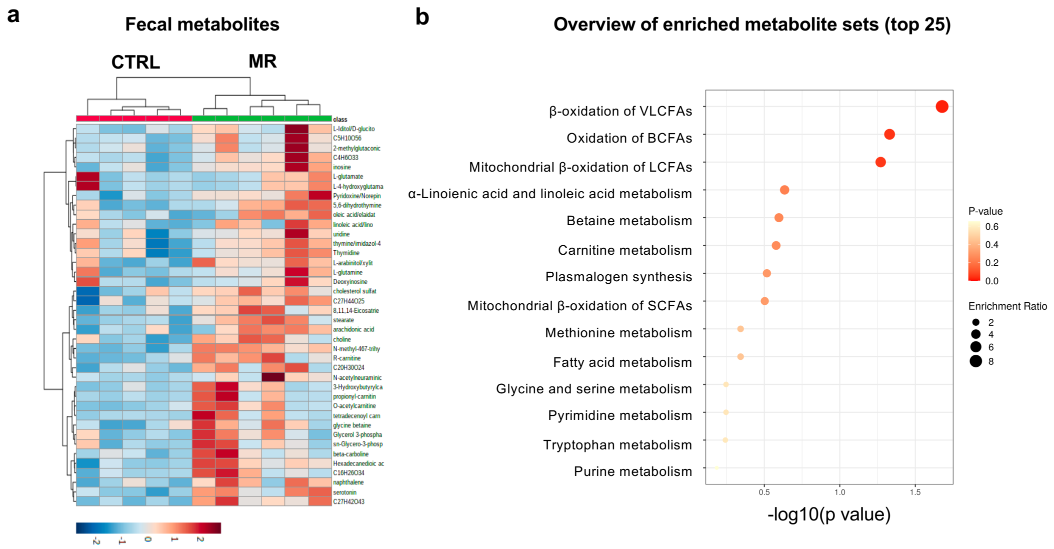

Supplementary Fig. 2. Dietary methionine restriction impacts fecal metabolites (Duke Platform).

a, C57BL/6J (B6) mice fed with different methionine diets have different fecal metabolites. Fecal metabolites from B6 fed with CTRL diet or MR diet were analyzed by LC-MS based metabolomics analysis by a Duke facility as described in Methods. A cluster analysis was performed using all significantly ($p<0.05$, $|FC|>1.5$) changed metabolites in MR group vs CTRL group. **b**, Dietary methionine restriction increases metabolites involved in fatty acid oxidation in feces of B6 mice. 19 upregulated fecal metabolites in MR fed mice were subjected for enrichment analysis using reference metabolome and SMPDB database in Metaboanalyst 5.0.

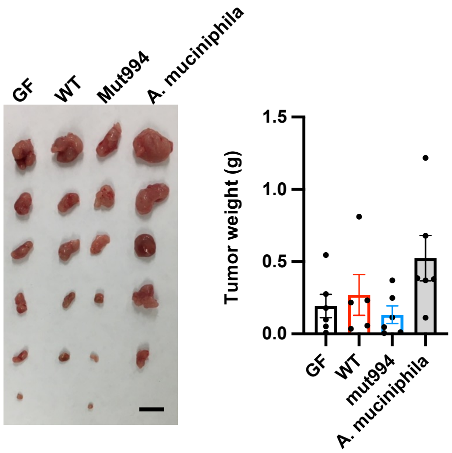

Supplementary Fig. 3. Mono-colonization of indicated bacteria in germ-free Balb/c does not significantly impact the growth of syngeneic CT26.CL25 tumors. Germ-free Balb/c mice (GF) were gavaged with indicated bacteria then inoculated with CT26.CL25 cells, and tumors were collected and analyzed as described in Methods (n=6, 5, 6, and 5 mice, with 1 injection site/mouse, for each indicated experimental condition, respectively). Values are expressed as mean ± s.e.m..

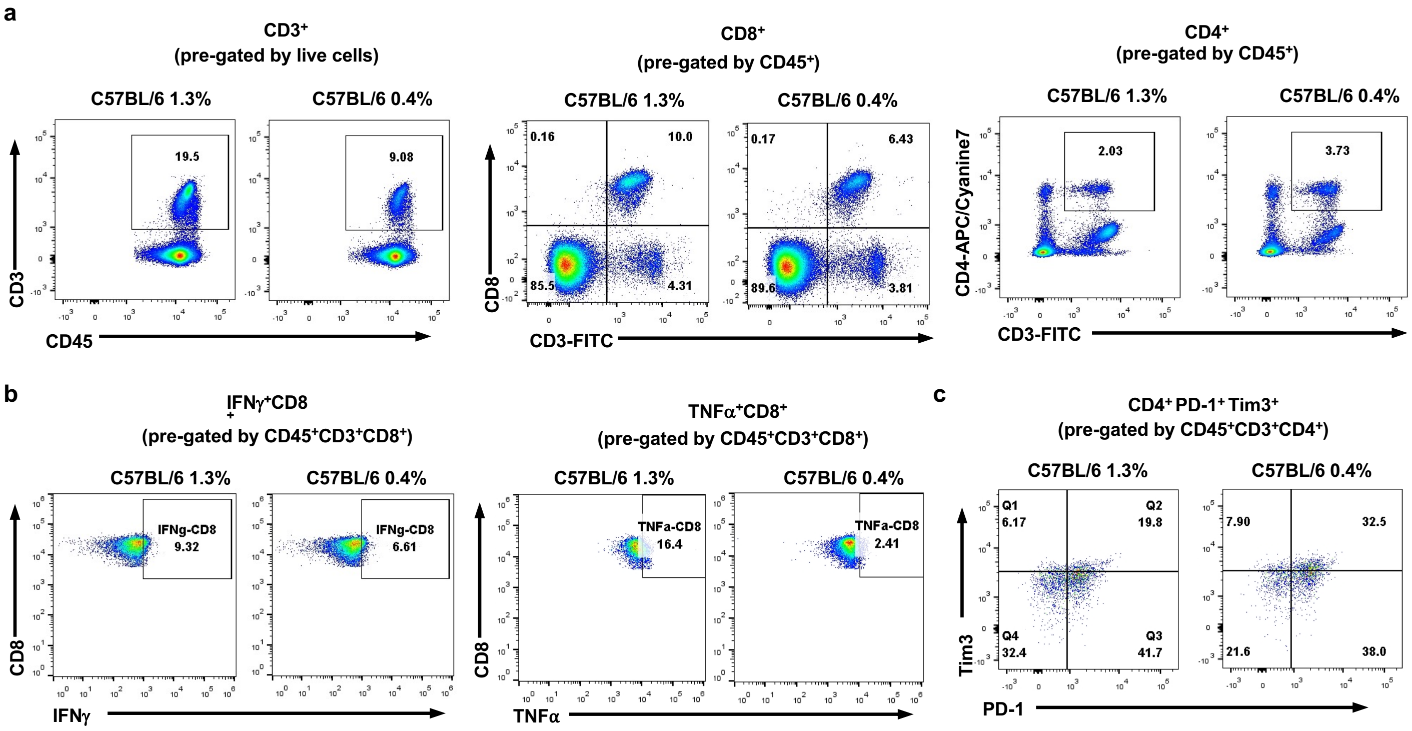

Supplementary Fig. 4. Dietary supplementation of methionine increases anti-tumor immunity in C57BL/6J mice.

Representative flow cytometry plots for indicated immune cells.

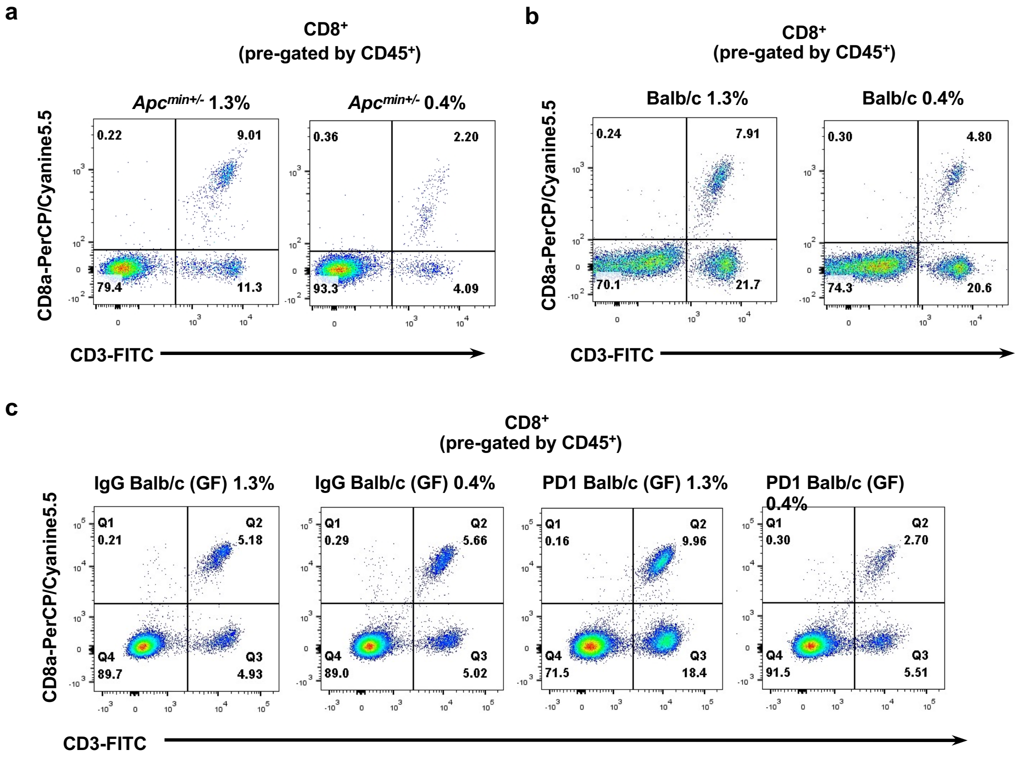

Supplementary Fig. 5. Dietary supplementation of methionine increases anti-tumor immunity in *Apc$^{min+/-}$* and Balb/c mice.

Representative flow cytometry plots for indicated immune cells.

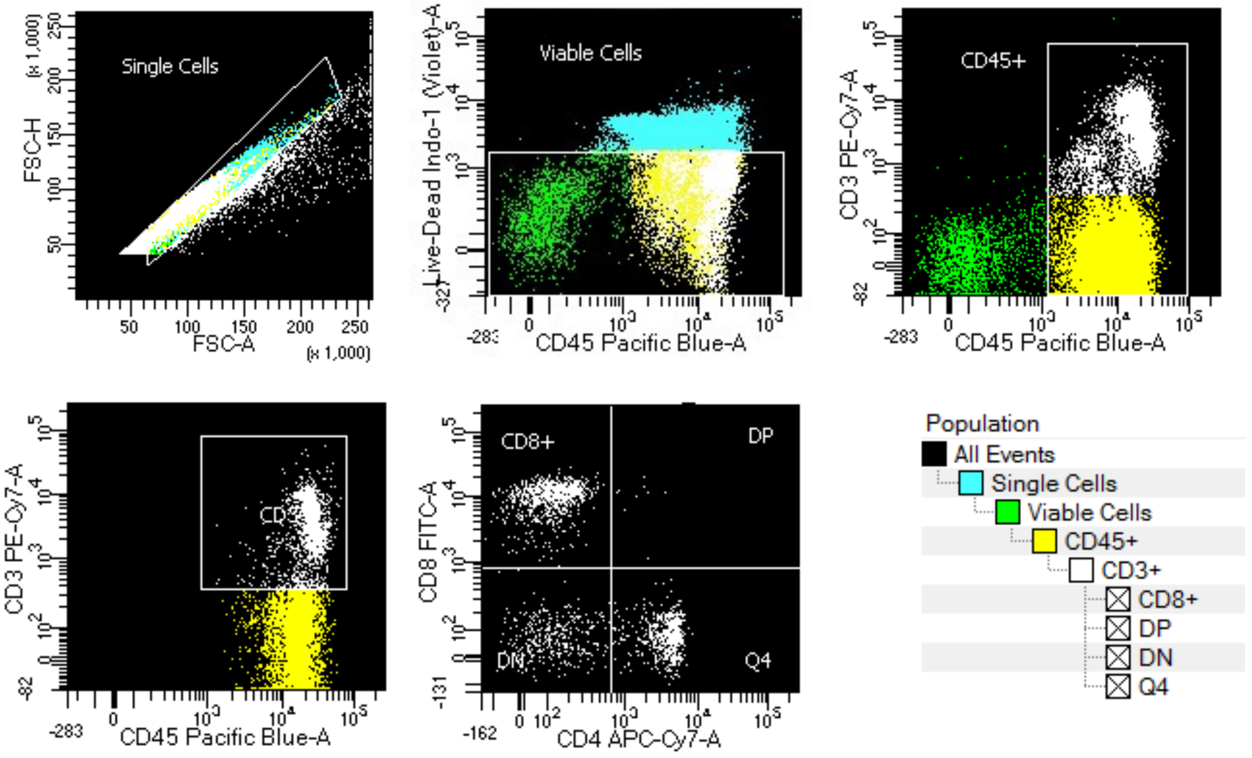

Supplementary Fig. 6. FACS gating strategy in this study.
