# Supplementary material for: Methionine restriction-induced sulfur deficiency impairs antitumour immunity partially through gut microbiota
Source: Nat Metab. 2023 Aug 3;5(9):1526–43. doi: 10.1038/s42255-023-00854-3 (PMC10513933; doi:10.1038/s42255-023-00854-3)
Supplement: Supplementary file 2 — Reporting Summary [file 42255_2023_854_MOESM2_ESM.pdf]

nature portfolio

Corresponding author(s): Xiaoling Li
Last updated by author(s): Jun 12, 2023

Reporting Summary

Nature Portfolio wishes to improve the reproducibility of the work that we publish. This form provides structure for consistency and transparency in reporting. For further information on Nature Portfolio policies, see our Editorial Policies and the Editorial Policy Checklist.

Statistics

For all statistical analyses, confirm that the following items are present in the figure legend, table legend, main text, or Methods section.

n/a	Confirmed	
☐	☒	The exact sample size (*n*) for each experimental group/condition, given as a discrete number and unit of measurement
☐	☒	A statement on whether measurements were taken from distinct samples or whether the same sample was measured repeatedly
☐	☒	The statistical test(s) used AND whether they are one- or two-sided<br>*Only common tests should be described solely by name; describe more complex techniques in the Methods section.*
☒	☐	A description of all covariates tested
☒	☐	A description of any assumptions or corrections, such as tests of normality and adjustment for multiple comparisons
☐	☒	A full description of the statistical parameters including central tendency (e.g. means) or other basic estimates (e.g. regression coefficient) AND variation (e.g. standard deviation) or associated estimates of uncertainty (e.g. confidence intervals)
☐	☒	For null hypothesis testing, the test statistic (e.g. *F*, *t*, *r*) with confidence intervals, effect sizes, degrees of freedom and *P* value noted<br>*Give P values as exact values whenever suitable.*
☒	☐	For Bayesian analysis, information on the choice of priors and Markov chain Monte Carlo settings
☒	☐	For hierarchical and complex designs, identification of the appropriate level for tests and full reporting of outcomes
☐	☒	Estimates of effect sizes (e.g. Cohen's *d*, Pearson's *r*), indicating how they were calculated

Our web collection on statistics for biologists contains articles on many of the points above.

Software and code

Policy information about availability of computer code

Data collection	Flow cytometry: BD LSRFortessa instrument (BD Biosciences)<br>Real-time PCR: BioRad CFX Manager<br>Immunoblotting: Odyssey imaging system<br>Seahorse: Wave software<br>RNA-seq: NextSeq500 (Illumina)<br>scRNA-seq library prepartion: 10x Genomics<br>scRNA-seq: NovaSeq6000 (Illumina)<br>Fecal and small intestinal bacterial 16S rRNA gene amplicon sequencing: MiSeq (Illumina)<br>Fecal bacterial RNA-seq (metatranscriptomics): NovaSeq6000 (Illumina)<br>Fecal metabolite analysis: LC-MS- Sieve 2.2 (Thermo Scientific)
Data analysis	Image quantification: ImageJ (Fiji).<br>Flow cytometry: FACSDiva (BD Biosciences) or FlowJo V10 (FlowJo LLC).<br>RNA-seq: STAR (Version 2.6) with Gencode vM23 annotation- Reads alignment; featureCount (subread, Version 1.4.6)- quantification; Bioconductor package DESeq2- DEG idenfication; Ingenuity Pathway Analysis (IPA, QIAGEN, USA; Content version 60467501) and GSEA software (Version 4.1)- Pathway enrichment analysis.<br>scRNA-seq: CellRanger 6.0.1 -preprocession the sequencing data; R version 4.0.1 - R environment for data analysis; Seurat_4.0.2 - R Data Class Environment used throughout analyses, SNN graph clustering implementation; celda_1.4.7- Remove ambient RNA; uwot_0.1.8- Creation of UMAP embeddings in R; harmony_l.0 - Batch correction for single cell data; MAST_1.16.0 - Used for calling marker gene and differential expression genes; pheatmap_1.0.12 - Data visualization; reticulate_1.20 - used for runing Python implementations within R; clusterProfiler_3.18.1 - preranked GSEA analysis of silenced or depressed genes; SingleCellExperiment_1.12.0- R Data Class Environment used

throughout analyses; https://biit.cs.ut.ee/gprofiler/gost- Pathway enrichment analysis.
Fecal bacterial 16S rRNA gene amplicon sequencing: Illumina Bcl2Fastq 2.18.0.12- Sequence conversion; QIIME 2 2018.11- Data processing and analysis.
Fecal bacterial RNA-seq (metatranscriptomics) analysis: fastp (v0.20.1)-reads trimming; bbduk (v38.84)-reads decontamination; kallisto index (v.0.46.2-k 15)-gene index; R DESeq2 package (v1.34.0)-differential expression analysis.
Fecal metabolite data processing: ProteoWizard (Version 3)-data conversion; XCMS (Version 3.16.1) in the IPO package (ver 1.20.0), which includes centWave, obiwarp, and density-parameter optimization; missForest package (ver.1.4), random forest algorithm-missing value imputation.
Fecal metabolite analysis: Sieve 2.2 (Thermo Scientific)-LC-MS peak extraction and integration; MetaboAnalyst4.0- Pathway analysis.
All other routine data analysis: Microsoft Excel and GraphPad Prism

For manuscripts utilizing custom algorithms or software that are central to the research but not yet described in published literature, software must be made available to editors and reviewers. We strongly encourage code deposition in a community repository (e.g. GitHub). See the Nature Portfolio guidelines for submitting code & software for further information.

Data

Policy information about availability of data

All manuscripts must include a data availability statement. This statement should provide the following information, where applicable:

- Accession codes, unique identifiers, or web links for publicly available datasets
- A description of any restrictions on data availability
- For clinical datasets or third party data, please ensure that the statement adheres to our policy

The RNA-seq dataset has been deposited to Gene Expression Omnibus under the accession number GSE165993.
The scRNA-seq dataset has been deposited to Gene Expression Omnibus under the accession number GSE181220.
The metatranscriptomic data has been submitted to the Sequence Read Archive under the accession number PRJNA892072.
Additional information about DEGs of RNA-seq data and IPA results is included in Supplementary Table 2 and 3.
The 16S rRNA amplicon sequencing results are in Supplementary Table 4.
The scRNA-seq results on immune cell clusters and T cell populations are in Supplementary Table 5, 6, and 7.
The fecal metabolites data are available in Supplementary Table 8.
The list of active transcripts of H2S producing enzymes in Supplementary Table 9.
All primers and antibodies used in the study are listed in Supplementary Table 10 and 11.
The raw data of all figures, including uncut immunoblots, are included in Source files.

Databases used for fecal metabolite annotation:
mzCloud Advanced Mass Spectral Database (mzCloud, Thermo Fisher Scientific) (https://www.mzcloud.org/); NIST 2020 LC-MS/MS library (NIST library, National Institute of Standards and Technology, MD) (https://www.nist.gov/programs-projects/nist20-updates-nist-tandem-and-electron-ionization-spectral-libraries); and ChemSpider chemical structure database (ChemSpider) (http://www.chemspider.com/).

Research involving human participants, their data, or biological material

Policy information about studies with human participants or human data. See also policy information about sex, gender (identity/presentation), and sexual orientation and race, ethnicity and racism.

Reporting on sex and gender	We analyzed the relationship between dietary protein intake and cancer risk using a subset of the existing UK Biobank data (UKB, https://www.ukbiobank.ac.uk/). This subset of data contains 31,626 total participants, including 16,462 (52%) females and 15,164 (48%) males (self-reported). Our analysis indicates that all and male participants in the low dietary protein intake group had significantly higher overall cancer risk compared to those in the high dietary protein intake group. In females, this difference is not significant. This information is provided in the Methods section and in Supplementary Table 1.
Reporting on race, ethnicity, or other socially relevant groupings	No such information is specified for our analysis.
Population characteristics	The age of participants at time of diet24 data collection was between 40 and 75, with a modal class (of most frequently seen ages) being between 55 and 70 years in both groups, i.e., with 'low protein' and with 'high protein' intake.
Recruitment	Only existing human data from the UKB were used in this study. No recruitment of participants.
Ethics oversight	The reference ID for Duke IRB Protocol of this study is Pro00109279.

Note that full information on the approval of the study protocol must also be provided in the manuscript.

Field-specific reporting

Please select the one below that is the best fit for your research. If you are not sure, read the appropriate sections before making your selection.

☒ Life sciences ☐ Behavioural & social sciences ☐ Ecological, evolutionary & environmental sciences

For a reference copy of the document with all sections, see nature.com/documents/nr-reporting-summary-flat.pdf

Life sciences study design

All studies must disclose on these points even when the disclosure is negative.

Sample size	For all allograft experiments, the sample size was estimated to achieve 30% of tumor size/weight difference with 80% of power. For all animal studies in Apcmin+/- mice, the sample size was estimated to achieve 2-fold difference of tumor number with 80% of power. For gene expression analysis, the sample size was estimated to achieve 2-fold difference of gene expression level with 80% of power. For in vitro cell culture experiments, no explicit calculations were done to determine the sample size. They were performed with at least 3 biological replicates and 2-tailed unpaired student's t-test was used to determine the significance of the difference.
Data exclusions	For all in vivo experiments, outlier samples that fall below Q1 − 3.0 IQR or above Q3 + 3.0 IQR were removed. There were marked in the source data files.
Replication	For all allograft experiments, 4-5 mice per group and 2 tumor injections/mouse were used in each independent experiment. Because not all injections resulted in tumor growth, so the final tumor number was 4-10/group. For tumor number/burden studies in Apcmin+/- mice, at least 10 mice/group were used in each independent experiment. Gene expression and FACS analysis were done with at least 4 mice/group. For scRNA-seq experiment, single cell suspension from 2 mice/group was combined for each sample. In vitro cell culture experiments were independently performed at least three times. All attempts at replication were successful with similar results. The replication number or mouse number for each experiment was indicated in the figure legends.
Randomization	Mice in all animal experiments were randomized to experimental groups.
Blinding	For omics data, the bioinformaticians were blinded to sample allocation in the initial analyses and sample processing steps. They were informed sample allocation for final statistical analyses. For other experiments, since prior knowledge is required for experimental set up, investigators were not blinded to experimental conditions.

Reporting for specific materials, systems and methods

We require information from authors about some types of materials, experimental systems and methods used in many studies. Here, indicate whether each material, system or method listed is relevant to your study. If you are not sure if a list item applies to your research, read the appropriate section before selecting a response.

Materials & experimental systems

n/a	Involved in the study	
☐	☒	Antibodies
☐	☒	Eukaryotic cell lines
☒	☐	Palaeontology and archaeology
☐	☒	Animals and other organisms
☒	☐	Clinical data
☒	☐	Dual use research of concern
☒	☐	Plants

Methods

n/a	Involved in the study	
☒	☐	ChIP-seq
☐	☒	Flow cytometry
☒	☐	MRI-based neuroimaging

Antibodies

Antibodies used

For flowcytometry, all antibodies were used at a 1:1000 dilution.
LIVE/DEAD™ Fixable Near-IR: Company: Invitrogen Catalog L;10119 Lot No. 1951432
anti-mouse CD16/32: Biolegend Clone:93 Catalog No: 101320 Lot No: B295040
anti-CD45-antibody-eFluor™ 450: Company: Invitrogen Clone: 30-F11 Catalog No. 48-0451-82 Lot No. 2423798
Anti-CD4-antibody-FITC: Company: Biolegend Clone: GK1.5 Catalog No: 100406 Lot No: B351029
Anti-CD4-antibody-APC/Cyanine7: Company: Biolegend Clone: RM4-5 Catalog No: 100525 Lot No: B351740
Anti-CD3-antibody-FITC: Company: Biolegend Clone: 17A2 Catalog No: 100204 Lot No: B351638
Anti-CD3-antibody-PE/Cyanine7: Company: Biolegend Clone: 17A2 Catalog No: 100220 Lot No: B356288
Anti-CD8-antibody-PerCP/Cyanine5.5: Company: Biolegend Clone: 53-6.7 Catalog No: 100734 Lot No: B292848
Anti-CD279 (PD-1)-antibody-PE: Company: Biolegend Clone: 29F.1A12 Catalog No: 135206 Lot No: B351740
Anti-CD366 (Tim-3)-antibody- Brilliant Violet 711™: Company: Biolegend Clone: RMT3-23 Catalog No: 119727 Lot No: B309627
anti-IFN gamma-antibody-APC: Company: Invitrogen Clone: XMG1.2 Catalog No. 17-7311-82 Lot No. 2175632
anti-TNF alpha-antibody-PE: Company: Invitrogen Clone: MP6-XT22 Catalog No. 12-7321-82 Lot No. 2124591
Rat IgG2b kappa Isotype Control-eFluor™ 450: Company: Invitrogen Clone: eB149/10H5 Catalog No. 48-0451-82 Lot No. 2423798
Rat IgG2b k Isotype Control-FITC: Company: Biolegend Clone: RTK4530 Catalog No: 400605 Lot No: B265825
Rat IgG2b k Isotype Control-PerCP/Cyanine5.5: Company: Biolegend Clone: RTK4530 Catalog No: 400631 Lot No: B327703
Rat IgG2b k Isotype Control-PE/Cyanine7: Company: Biolegend Clone: RTK4530 Catalog No: 400617 Lot No: B334374
Rat Ig2a k Isotype Control-APC: Company: BD Clone: R35-95 Catalog No: 553932 Lot No: 1165045

The following antibodies were used for in vivo treatment:
anti-PD-1 blocking antibody: Company: BioXcell Clone: RMP1-14 Catalog No: BE0146 (200 or 300 μg per mouse)
InVivoMAb rat IgG2a isotype control: Company: BioXcell Clone: 2A3Catalog No: BE0089 Lot No: 849322J2 (200 or 300 μg per mouse)

	For western blot:<br>Bax-antibody: Company: Cell signaling Clone:87G3 Catalog No: cs2772 1:1000 dilution<br>Actin-antibody: Company: Millipore Sigma Clone:48H2 Catalog No: 9197 1:10,000 dilution<br>GAPDH-antibody: Company: Millipore Sigma Clone:13E5 Catalog No: 5125 1:2000 dilution
Validation	All these antibodies are routinely used for FACS and immunoblotting, and have been cited by many publications. They are all validated by manufacturers, mainly eBioscience and Biolegend. Validation materials are available on respective manufacturer home pages for each antibody.

Eukaryotic cell lines

Policy information about cell lines and Sex and Gender in Research

Cell line source(s)	CT26.WT (ATCC CRL-2638), CT26.CL25 (ATCC CRL-2639), B16.F10 (ATCC CRL-6475) were purchased from ATCC.
Authentication	The authentication of all above murine cell lines was performed by ATCC cell line authentication service using short tandem repeat (STR) profiling in 2022.
Mycoplasma contamination	All cell lines were tested negative for the mycoplasma contamination by the Quality Assurance Laboratory of the National Institute of Environmental Health Sciences.
Commonly misidentified lines<br>(See ICLAC register)	No commonly misidentified lines were used.

Animals and other research organisms

Policy information about studies involving animals; ARRIVE guidelines recommended for reporting animal research, and Sex and Gender in Research

Laboratory animals	Mouse, Apcmin+/- (Jax: 002020), males and females, adults (staring from 6-8 weeks old).<br>Mouse, Apcmin+/- /Rag2-/-, males and females, adults (staring from 6-8 weeks old).<br>Mouse, C57BL/6J (Jax: 000664), males and females, adults (staring from 6-8 weeks old).<br>Mouse, BALB/cJ (Jax: 000651), males, adults (staring from 6-8 weeks old).<br>Mouse, NSG (Jax: 005557), males, adults (staring from 6-8 weeks old).
Wild animals	The study did not involve wild animals.
Reporting on sex	A few studies using Apcmin+/- mice were performed using both sexes (Fig. 1a, 5b, 8f, and ED Fig. 2g). Both males and females displayed similar responses to dietary treatments. We provided disaggregated data for sex in the corresponding source data files.<br><br>The AOM/DSS CRC procedure was performed in both sexes, and consistent results on body weight and survival were obtained and reported as source data for Fig. 1d. The final tumor results in Fig. 1e were from females, because 75% of MR-diet fed male mice underwent this procedure died or were removed due to healthy concerns before reaching the experimental endpoint.<br><br>All experiments with syngeneic cancer models were performed only in male mice, primarily because the observations from above two cancer models showed no sex-based difference for this study.
Field-collected samples	The study did not involve samples collected from the field.
Ethics oversight	All animal work was approved by the Institutional Animal Care and Use Committee of the National Institute of Environmental Health Sciences under the Animal Study Proposal numbers ASP2014-0016 and ASP2017-0012.

Note that full information on the approval of the study protocol must also be provided in the manuscript.

Flow Cytometry

Plots

Confirm that:

☒ The axis labels state the marker and fluorochrome used (e.g. CD4-FITC).

☒ The axis scales are clearly visible. Include numbers along axes only for bottom left plot of group (a 'group' is an analysis of identical markers).

☒ All plots are contour plots with outliers or pseudocolor plots.

☒ A numerical value for number of cells or percentage (with statistics) is provided.

Methodology

Sample preparation	Blood samples were collected with EDTA coated tubes. Red blood cells were lysed with ACK lysis buffer at room temperature for 10 min. The collected lymphocytes were incubated with anti-mouse CD16/32 at room temperature for 10 min to block the IgG Fc receptors. Expression of surface markers was detected by simultaneously staining with the following antibodies

(eFluor 450 anti-mouse CD45, APC-Cy7 anti-mouse CD4, FITC anti-mouse CD4, FITC anti-mouse CD3, PE-Cy7 anti-mouse CD3, FITC anti-mouse CD8, PerCP-Cy™5.5 anti-mouse CD8, PerCP-Cy5.5 PE anti-mouse CD279 (PD-1), BV711 anti-mouse Tim3) and LIVE/DEAD fixable Aqua dead cell stain kit (ThermoFisher, Cat. # L34957) at room temperature for 15 min followed by flow cytometry.

For the IFNγ and TNFα staining, after perform cell surface staining, the cells were first fixed using BD Cytofix ™ solution (Cat. # 554655) then permeabilized using BD Perm/Wash buffer (Cat. # 554723). Add 2 ml PBS to wash cells once. Add 1 ml the True-Nuclear™ 1x Perm Buffer to each tube, centrifuge tubes at 400 g at room temperature for 5 minutes and discard the supernatant. Resuspend the cell pellet in 100 µL of 1x Perm buffer with antibody for 30 minutes in the dark. Add 1 ml of 1x Perm buffer to each tube. Centrifuge the tubes at 400g at room temperature for 5 minutes and discard the supernatant. Add 1 ml cell staining buffer (Biolegend, Cat. # 420201) to each tube. Centrifuge the tubes at 400g at room temperature for 5 minutes and discard the supernatant. Resuspend in 0.3ml cell staining buffer, and then acquire the tubes on a flow cytometer.

Instrument	Flow cytometric analysis was performed on BD LSRFortessa instrument (BD Biosciences).
Software	FACSDiva (BD Biosciences) or FlowJo V10 (FlowJo LLC) software.
Cell population abundance	Initial forward scatter versus side-scatter gates were carefully adjusted by backgating on live CD45+ populations to include all cells and exclude debris. Stringent exclusion of dead cells and doublets was performed before gating for immune cells (CD45 +) .
Gating strategy	T cells were gated by live CD45+CD3+ and subsetted into CD4 T (CD4+) cells or CD8 T (CD8+) cells; CD4 T cells were further subsetted as Treg cells (CD4+CD25+FOXP3+) or exhausted CD4+ T cells (CD4+Tim3+PD1+); CD8 T cells were further subsetted as the IFNγ expressing CD8+ T cells (CD8+ IFNγ+).

☒ Tick this box to confirm that a figure exemplifying the gating strategy is provided in the Supplementary Information.
