# Supplementary figures and images for: Methionine restriction-induced sulfur deficiency impairs antitumour immunity partially through gut microbiota
Source: Nat Metab. 2023 Aug 3;5(9):1526–43. doi: 10.1038/s42255-023-00854-3 (PMC10513933; doi:10.1038/s42255-023-00854-3)

f

# GAPDH sulfhydrylation

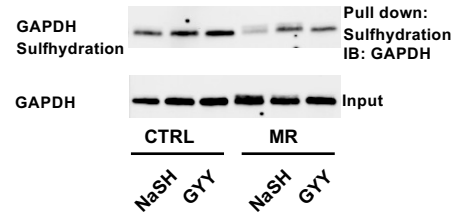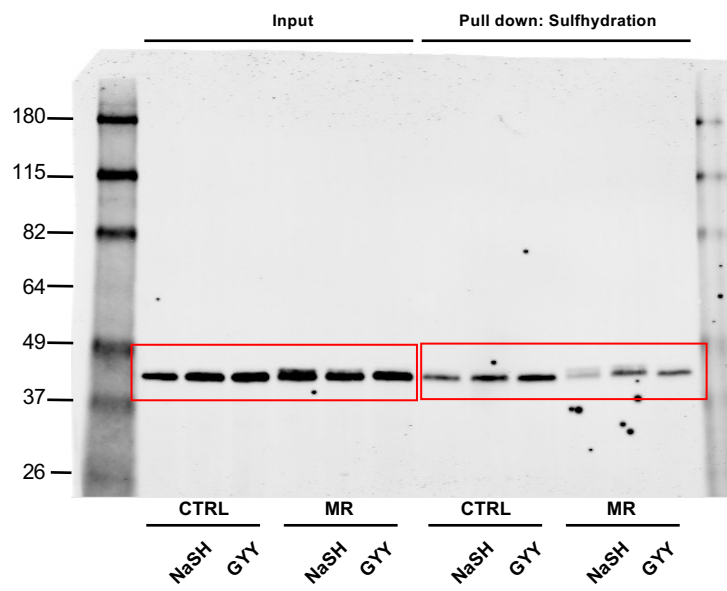

Raw uncut gel images for Fig. 6f.

Supplement: Supplementary file 11 — Unprocessed immunoblots for Fig. 6f. [file 42255_2023_854_MOESM11_ESM.pdf]

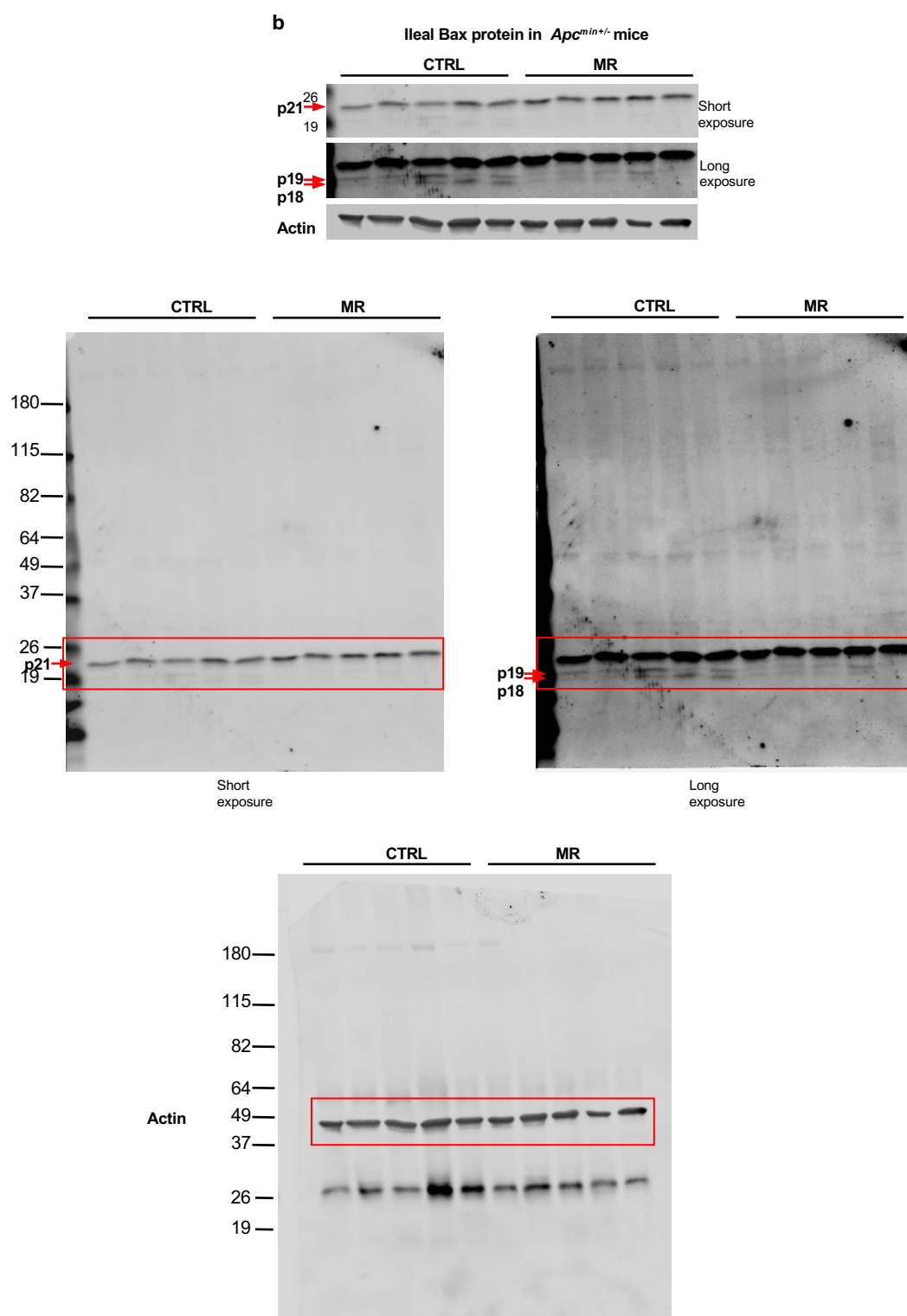

Source file-Extended Data Fig. 1b Raw uncut gel images

Supplement: Supplementary file 15 — Unprocessed immunoblots for Extended Data Fig. 1b. [file 42255_2023_854_MOESM15_ESM.pdf]
